# Supplementary material for: The influence of garden spatial configuration on tourist behavior: A systematic review based on Space Syntax
Source: PLoS One. 2026 Jan 2;21(1):e0339994. doi: 10.1371/journal.pone.0339994 (PMC12758741; doi:10.1371/journal.pone.0339994)
Supplement: S1 Table — (PDF) [file pone.0339994.s001.pdf]

S1 Table. Detailed search strategy

Database	Strategy	Results
Web of Science	(ALL=((“garden” OR “park” OR “grove”) AND ((“space” AND “syntax”) OR (“spatial” AND “syntax”))	180
SCOPUS	(ALL=(“garden” OR “park” OR “grove” (“space” AND “syntax”)OR(“spatial” AND “syntax”)	156
JSTOR	("garden" OR "park" OR "grove") AND ("space syntax" OR "spatial syntax")	58
ScienceDirect	("garden" OR "park" OR "grove") AND ("space syntax" OR "spatial syntax")	646
