# Supplementary material for: The influence of garden spatial configuration on tourist behavior: A systematic review based on Space Syntax
Source: PLoS One. 2026 Jan 2;21(1):e0339994. doi: 10.1371/journal.pone.0339994 (PMC12758741; doi:10.1371/journal.pone.0339994)
Supplement: S2 Table — (PDF) [file pone.0339994.s002.pdf]

Supplementary Table 1: Crowe Critical Appraisal Tool (CCAT) form			
**Category item**	**Item descriptors**	**Description**	**Score (1-5)**
	**1. Preliminaries**		
Title	1. Includes study aims and designs		
**Abstract**	1. Key information<br>2. Balanced and informative		
Last	1. Sufficient detail others could reproduce<br>2. Clear/concise writing, table(s), diagram(s) and figure(s)		
		**Preliminaries (/5)**	
	**2. Introduction**		
Background	1. Summary of current knowledge<br>2. Specific problem(s) addressed and reason(s) for addressing		
Objective	1. Primary objective(s), hypothesis(es), or aim(s)<br>2. Secondary question(s)		
	**Is it worth continuing?**	**Introduction (/5)**	
	**3. Design**		
Research design	1. Research design was chosen and why<br>2. Suitability of research design(s)		
Intervention, treatment, exposure	1. Intervention(s)/ treatment(s)/ exposure(s) chosen and why<br>2. Precise details of intervention(s)/ treatment(s)/ exposure(s) for each group<br>3. Intervention(s)/ treatment(s)/ exposure(s) valid and reliable		

Information Classification: General

The outcome, output, predictor, measure	1. Outcome(s)/ output(s)/ predictor(s)/ measure(s) chosen and why<br>2. Clearly define outcome(s)/ output(s)/ predictor(s)/ measure(s)<br>3. Outcome(s)/ output(s)/ predictor(s)/ measure(s) valid and reliable	
Bias, etc	1. Potential bias, confounding variables, effect modifiers, interactions<br>2. Sequence generation, group allocation, group balance, and by whom<br>3. Equivalent treatment of participants/ cases/ groups	
	**Is it worth continuing?**	**Design (/5)**
	**4. Sampling**	
Sampling method	1. Sampling method(s) chosen and why<br>2. Suitability of sampling method	
Sampling size	1. Sampling size, how chosen, and why<br>2. Suitability of sample size	
Sampling protocol	1. Target/actual/sample population(s): description and suitability<br>2. Participants/cases/groups: inclusion and exclusion criteria<br>3. Recruitment of participants/cases/groups	
	**Is it worth continuing?**	**Sampling (/5)**
	**5. Data collection**	
Collection method	1. Collection method(s) chosen and why<br>2. Suitability of collection method(s)	
Collection protocol	1. Include date(s), location(s), setting(s), personnel, material(s), process(es)<br>2. Methods to ensure/ enhance the quality of measurement/ instrumentation<br>3. Manage non-participation, withdrawal, incomplete/ lost data	
	**Is it worth continuing?**	**Data collection (/5)**
	**6. Ethical matters**	
Participant ethics	1. Informed consent, equity<br>2. Privacy, confidentiality/ anonymity	

Information Classification: General

Researcher ethics	1. Ethical approval, funding, conflict(s) of interest<br>2. Subjectivities, relationship(s) with participants/ cases	
	**Is it worth continuing?**	**Ethical matters (/5)**
	**7. Results**	
Analysis, Integration, Interpretation method	1. A.I.I. method(s) for primary outcome(s)/ output(s)/ predictor(s) chosen and why<br>2. Additional A.I.I. methods (e.g., subgroup analysis) chosen and why<br>3. Suitability of analysis/ integration/ interpretation method	
Essential analysis	1. Flow of participants/ cases/ groups through each stage of research<br>2. Demographic and other characteristics of participants/ cases/ groups<br>3. Analyze raw data, response rate, non-participation/ withdrawal/ incomplete/lost data	
The outcome, output, predictor analysis	1. Summary of results and precision for each outcome/ output/ predictor/ measure<br>2. Consideration of benefits/ harms, unexpected results, problems/ failures<br>3. Description of outlying data (e.g., diverse cases, adverse effects, minor themes)	
		**Results (/5)**
	**8. Discussion**	
Interpretation	1. Interpretation of results in the context of current evidence and objectives<br>2. Draw inferences consistent with the strength of the data<br>3. Consideration of alternative explanations for observed results<br>4. Account for bias, confounding/ effect modifiers/ interactions/ imprecision	
Generalization	1. Consideration of the overall practical usefulness of the study<br>2. Description of generalizability (external validity) of the study	

Information Classification: General

Concluding remarks	1. Highlight the study’s particular strength<br>2. Suggest steps that may improve future results (e.g., limitations)<br>3. Suggest further studies	
		**Discussion (/5)**
	**9. Total**	
Total score	1. Add all scores for categories 1-8	

Note: Scoring for each category is based on the guiding principles recommended in the Crowe Critical
Crowe Critical Appraisal Tool (CCAT): Version 1.4 (19 November 2013): Michael Crowe (michael.crowe@my.jcu.edu.au)

Information Classification: General

Supplementary Table 2: Critical appraisal of included studies.										
Scores										
Category Items		1) Preliminaries	2) Introduction	3) Design	4) Sampling	5) Data Collection	6) Ethical matters (participant ethics and researcher ethics)	7) Results	8) Discussion	9) Aggregate Scores (/40)
1	Sharma[29] (2022) India	5	5	4	3	3	4	5	4	33
2	Kirikkanat [46] (2018) Turkey	5	5	4	4	4	4	5	5	36
3	Noviati [32] (2017) Indonesia	5	5	3	3	4	5	5	4	34
4	Jafri [6] (2017) India	5	5	5	4	4	5	5	5	38
5	Sava [60] (2020) Romania	5	5	5	5	5	4	4	4	37
6	Adil [47] (2020) Pakistan	5	5	4	4	4	4	5	5	36
7	Lisnyj [96] (2022)	5	5	4	4	4	4	5	5	36

Information Classification: General

	Canada									
8	Poots [11] (2020) United Kingdom	5	5	4	3	4	4	5	4	34
9	Li [54] (2022) China	5	5	4	4	3	4	5	5	35
10	Ramirez-Perez [85] (2022) Chile	5	5	5	4	4	4	5	5	37
11	Liu [95] (2015) China	5	5	4	4	5	3	4	5	35
12	Raza [80] (2020) Pakistan	5	5	4	5	5	5	5	4	38
13	Virga [10] (2022) Romania	5	5	4	4	5	3	5	4	35
14	Zhang [55] (2020) China	5	5	3	3	3	4	5	4	32
15	Liran[67] (2019) Israel	5	5	4	4	4	4	5	5	36
16	Slatten [57] (2023) Norway	5	5	5	4	4	5	4	4	36
17	Adil [47] (2021) Pakistan	5	5	4	4	4	4	4	4	34
18	Luthans [61]	5	5	3	3	3	4	5	4	32

	(2022) USA									
19	da Costa [52] (2021) Portugal	5	5	3	3	3	4	5	5	33
20	You [71] (2016) Korea	5	5	5	5	4	4	5	5	38
21	Hicks [50] (2015) Australia	5	5	3	3	3	4	4	4	31
22	Geremias [84] (2022) Portugal	5	5	3	3	3	4	4	5	32
23	Saman [68] (2021) Indonesia	5	5	3	4	4	4	5	5	35
24	Xu [86] (2022) China	5	5	3	3	3	4	4	4	31
25	Sweet [63] (2020) USA	5	5	3	3	3	4	5	4	32
26	Chaffin [89] (2023) USA	5	5	4	4	4	4	5	5	36
27	Lin [74] (2020) Taiwan	5	5	3	3	3	4	4	5	32
28	Chen [87] (2023) Taiwan	5	5	4	4	4	4	5	5	36

Information Classification: General

29	Qiu [56] (2023) China	5	5	5	5	5	4	4	5	38
30	Sanchez-Cardona [90] (2021) USA	5	5	4	4	5	5	4	5	37
31	Rad [75] (2017) Iran	5	5	4	5	4	4	5	4	36
32	Luthans [33] (2016) USA	5	5	5	5	4	4	4	5	37
33	Ortega-Maldonado [59] (2018) Spain Spain	5	5	4	5	4	4	4	5	36
34	Wang [76] (2021) China	5	5	4	5	4	4	4	5	36
35	Luthans [24] (2019) USA	5	5	5	4	4	4	4	5	36
36	Chua [66] (2018) Malaysia	5	5	3	3	3	4	5	4	32
37	Martinez [8] (2019) Spain/Portugal	5	5	3	3	3	4	4	5	32
38	Lye [51] (2022) Malaysia	5	5	4	4	4	4	5	5	36

Information Classification: General

39	Nambudiri [23]<br>(2020)<br>India	5	5	3	3	3	4	5	4	32
40	Geremias [26]<br>(2020)<br>Portugal	5	5	4	4	5	4	5	5	37
41	Yu [77]<br>(2021)<br>China	5	5	4	4	5	5	5	5	38
42	Geremias [91]<br>(2021)<br>Portugal	5	5	3	3	3	4	4	4	31
43	Radack [78]<br>(2022)<br>USA	5	5	4	4	4	5	5	5	37

Information Classification: General
