# Supplementary material for: The influence of garden spatial configuration on tourist behavior: A systematic review based on Space Syntax
Source: PLoS One. 2026 Jan 2;21(1):e0339994. doi: 10.1371/journal.pone.0339994 (PMC12758741; doi:10.1371/journal.pone.0339994)
Supplement: S4 Table — (PDF) [file pone.0339994.s004.pdf]

S4 Table. Numbered table of all studies

No	Title	Included in Analysis	Reason for Exclusion
1.	The relationship between the distribution and use patterns of parks and their spatial accessibility at the city level: A case study from Tehran, Iran	NO	Duplicate records
2.	The relationship between the distribution and use patterns of parks and their spatial accessibility at the city level: A case study from Tehran, Iran	NO	Duplicate records
3.	The relationship between the distribution and use patterns of parks and their spatial accessibility at the city level: A case study from Tehran, Iran	NO	Duplicate records
4.	Analyzing urban parks' spatial integration in Budapest to understand changes in visitation patterns during the COVID-19 pandemic	NO	Duplicate records
5.	Analyzing urban parks' spatial integration in Budapest to understand changes in visitation patterns during the COVID-19 pandemic	NO	Duplicate records
6.	Association between park vitality and commercial vitality: a case study in Chengdu	NO	Duplicate records
7.	Association between park vitality and commercial vitality: a case study in Chengdu	NO	Duplicate records
8.	Measuring pedestrian-level street greenery visibility through space syntax and crowdsourced imagery: A case study in London, UK	NO	Duplicate records
9.	Measuring pedestrian-level street greenery visibility through space syntax and crowdsourced imagery: A case study in London, UK	NO	Duplicate records
10.	Metric or topological proximity? The associations among proximity to parks, the frequency of residents' visits to parks, and perceived stress	NO	Duplicate records
11.	Metric or topological proximity? The associations among proximity to parks, the frequency of residents' visits to parks, and perceived stress	NO	Duplicate records
12.	THERMAL COMFORT, VISIBILITY, AND THE SPATIAL LAYOUT IN CLASSICAL GARDENS OF SUZHOU, CHINA	NO	Duplicate records
13.	THERMAL COMFORT, VISIBILITY, AND THE SPATIAL LAYOUT IN CLASSICAL GARDENS OF SUZHOU, CHINA	NO	Duplicate records
14.	Evaluating cultural ecosystem services in China's modern historic parks: A sentiment computing approach	NO	Duplicate records
15.	Evaluating cultural ecosystem services in China's modern historic parks: A sentiment computing approach	NO	Duplicate records
16.	Optimisation of Ecological Leisure Industrial Planning Based on Improved GIS-AHP: A Case Study in Shapingba District, Chongqing, China	NO	Duplicate records
17.	Optimisation of Ecological Leisure Industrial Planning Based on Improved GIS-AHP: A Case Study in Shapingba District, Chongqing, China	NO	Duplicate records
18.	A framework to evaluate the accessibility, visibility, and intelligibility of green-blue spaces (GBSs) related to pedestrian movement	NO	Duplicate records
19.	A framework to evaluate the accessibility, visibility, and intelligibility of green-blue spaces (GBSs) related to pedestrian movement	NO	Duplicate records
20.	Mapping Rectangles to Cylinders: Waste-Conscious Constructions of Curved Structural Surfaces	NO	Duplicate records
21.	Mapping Rectangles to Cylinders: Waste-Conscious Constructions of Curved Structural Surfaces	NO	Duplicate records
22.	Space Syntax with Logic Programming: An Application to a Modern Estate	NO	Duplicate records
23.	Space Syntax with Logic Programming: An Application to a Modern Estate	NO	Duplicate records
24.	Evolution and Influencing Factors of Township Spatial Form: A Two-Dimensional Perspective	NO	Duplicate records
25.	Evolution and Influencing Factors of Township Spatial Form: A Two-Dimensional Perspective	NO	Duplicate records
26.	House prices and relative location	NO	Duplicate records
27.	House prices and relative location	NO	Duplicate records
28.	House prices and relative location	NO	Duplicate records
29.	Planning a Green Infrastructure Network to Integrate Potential Evacuation Routes and the Urban Green Space in a Coastal City: The Case Study of Haeundae District, Busan, South Korea	NO	Duplicate records
30.	Planning a Green Infrastructure Network to Integrate Potential Evacuation Routes and	NO	Duplicate records

	the Urban Green Space in a Coastal City: The Case Study of Haeundae District, Busan, South Korea		
31.	Planning a Green Infrastructure Network to Integrate Potential Evacuation Routes and the Urban Green Space in a Coastal City: The Case Study of Haeundae District, Busan, South Korea	NO	Duplicate records
32.	External Spatial Morphology of Creative Industries Parks in the Industrial Heritage Category Based on Spatial Syntax: Taking Tianjin as an Example	NO	Duplicate records
33.	External Spatial Morphology of Creative Industries Parks in the Industrial Heritage Category Based on Spatial Syntax: Taking Tianjin as an Example	NO	Duplicate records
34.	Sensing perceived urban stress using space syntactical and urban building density data: A machine learning-based approach	NO	Duplicate records
35.	Sensing perceived urban stress using space syntactical and urban building density data: A machine learning-based approach	NO	Duplicate records
36.	Sensing perceived urban stress using space syntactical and urban building density data: A machine learning-based approach	NO	Duplicate records
37.	Research on the renewal and renovation of Rabbit Mountain Site Park oriented by the construction of green blocks	NO	Duplicate records
38.	Research on the renewal and renovation of Rabbit Mountain Site Park oriented by the construction of green blocks	NO	Duplicate records
39.	The six dimensions of built environment on urban vitality: Fusion evidence from multi-source data	NO	Duplicate records
40.	The six dimensions of built environment on urban vitality: Fusion evidence from multi-source data	NO	Duplicate records
41.	Interpreting the space characteristics of everyday heritage gardens of Suzhou, China, through a spacce syntax approach	NO	Duplicate records
42.	Interpreting the space characteristics of everyday heritage gardens of Suzhou, China, through a spacce syntax approach	NO	Duplicate records
43.	Analysis of Urban Park Accessibility Based on Space Syntax: Take the Urban Area of Changsha City as an Example	NO	Duplicate records
44.	Analysis of Urban Park Accessibility Based on Space Syntax: Take the Urban Area of Changsha City as an Example	NO	Duplicate records
45.	Spatial equity analysis of urban green space based on spatial design network analysis (sDNA): A case study of central Jinan, China	NO	Duplicate records
46.	Spatial equity analysis of urban green space based on spatial design network analysis (sDNA): A case study of central Jinan, China	NO	Duplicate records
47.	Seasonal effects on blue-green space preferences: examining spatial configuration and residents' perspectives	NO	Duplicate records
48.	Intelligent smart city parking facility layout optimization based on intelligent IoT analysis	NO	Duplicate records
49.	Intelligent smart city parking facility layout optimization based on intelligent IoT analysis	NO	Duplicate records
50.	Planting design for urban parks: Space syntax as a landscape design assessment tool	NO	Duplicate records
51.	Planting design for urban parks: Space syntax as a landscape design assessment tool	NO	Duplicate records
52.	Maximizing Public and Private Satisfaction for a Better Privately Owned Public Space: The Case of Yeouido Business District	NO	Duplicate records
53.	Maximizing Public and Private Satisfaction for a Better Privately Owned Public Space: The Case of Yeouido Business District	NO	Duplicate records
54.	Prediction of Human Movement in Open Public Spaces: Case Study of Sarajevo	NO	Duplicate records
55.	Prediction of Human Movement in Open Public Spaces: Case Study of Sarajevo	NO	Duplicate records
56.	Pandemic-Resilient Urban Centers: A New Way of Thinking for Industrial-Oriented Urbanization in Ethiopia	NO	Duplicate records
57.	Pandemic-Resilient Urban Centers: A New Way of Thinking for Industrial-Oriented Urbanization in Ethiopia	NO	Duplicate records
58.	Transport infrastructure modifications and accessibility to public parks in Greater Cairo	NO	Duplicate records
59.	Transport infrastructure modifications and accessibility to public parks in Greater Cairo	NO	Duplicate records
60.	Transport infrastructure modifications and accessibility to public parks in Greater Cairo	NO	Duplicate records
61.	Transport infrastructure modifications and accessibility to public parks in Greater Cairo	NO	Duplicate records

62.	Engaging in social interaction: relationships between the accessibility of path structure and intensity of passive social interaction in urban parks	NO	Duplicate records
63.	Engaging in social interaction: relationships between the accessibility of path structure and intensity of passive social interaction in urban parks	NO	Duplicate records
64.	The Constructed Instant: Gerrit Rietveld's Forgotten Sonsbeek Pavilion of 1958	NO	Duplicate records
65.	The Constructed Instant: Gerrit Rietveld's Forgotten Sonsbeek Pavilion of 1958	NO	Duplicate records
66.	Physiological responses to urban design during bicycling: A naturalistic investigation	NO	Duplicate records
67.	Physiological responses to urban design during bicycling: A naturalistic investigation	NO	Duplicate records
68.	Locating pocket parks: Assessing the effects of land use and accessibility on the public presence	NO	Duplicate records
69.	Locating pocket parks: Assessing the effects of land use and accessibility on the public presence	NO	Duplicate records
70.	Syntactical analysis of the accessibility and sociability of a square in the Kuala Lumpur City Center	NO	Duplicate records
71	Syntactical analysis of the accessibility and sociability of a square in the Kuala Lumpur City Center	NO	Duplicate records
72	Accessibility indicator for a trails network in a Nature Park as part of the environmental assessment framework	NO	Duplicate records
73	Accessibility indicator for a trails network in a Nature Park as part of the environmental assessment framework	NO	Duplicate records
74	Accessibility indicator for a trails network in a Nature Park as part of the environmental assessment framework	NO	Duplicate records
75	Effect of Space Configurational Attributes on Social Interactions in Urban Parks	NO	Duplicate records
76	Effect of Space Configurational Attributes on Social Interactions in Urban Parks	NO	Duplicate records
77	The Relationship between the Spatial Configuration and the Fourth Sustainable Dimension Creativity in University Campuses: The Case Study of Zernike Campus, Groningen, The Netherlands	NO	Duplicate records
78	The Relationship between the Spatial Configuration and the Fourth Sustainable Dimension Creativity in University Campuses: The Case Study of Zernike Campus, Groningen, The Netherlands	NO	Duplicate records
79	A new method to explore the abnormal space of urban hidden dangers under epidemic outbreak and its prevention and control: A case study of Jinan City	NO	Duplicate records
80	A new method to explore the abnormal space of urban hidden dangers under epidemic outbreak and its prevention and control: A case study of Jinan City	NO	Duplicate records
81	Accessibility of green spaces in a metropolitan network using space syntax to objectively evaluate the spatial locations of parks and promenades in Doha, State of Qatar	NO	Duplicate records
82	Accessibility of green spaces in a metropolitan network using space syntax to objectively evaluate the spatial locations of parks and promenades in Doha, State of Qatar	NO	Duplicate records
83	Accessibility of green spaces in a metropolitan network using space syntax to objectively evaluate the spatial locations of parks and promenades in Doha, State of Qatar	NO	Duplicate records
84	Outdoor Space Quality Mapping by Combining Accessibility, Openness, and Microclimate: A Case Study in a Neighborhood Park in Shanghai, China	NO	Duplicate records
85	Outdoor Space Quality Mapping by Combining Accessibility, Openness, and Microclimate: A Case Study in a Neighborhood Park in Shanghai, China	NO	Duplicate records
86	Approach to comprehensive analysis of bastion castle ensemble with historic gardens: novel combined strategy	NO	Duplicate records
87	Approach to comprehensive analysis of bastion castle ensemble with historic gardens: novel combined strategy	NO	Duplicate records
88	How does urban green space feature influence physical activity diversity in high-density built environment? An on-site observational study	NO	Duplicate records
89	How does urban green space feature influence physical activity diversity in high-density built environment? An on-site observational study	NO	Duplicate records
90	Spatial configuration analysis of a traditional garden in Yangzhou city: a comparative case study of three typical gardens	NO	Duplicate records
91	Optimization of spatial distribution of sports parks based on accessibility analysis	NO	Duplicate records
92	Optimization of spatial distribution of sports parks based on accessibility analysis	NO	Duplicate records
93	A tailored space syntax approach to the preservation and development of a cultural park	NO	Duplicate records

94	Integrating space syntax with field observations to understand the spatial logic of park infrastructure	NO	Duplicate records
95	Influential factors of spatial performance in metro-led urban underground public space: A case study in Shanghai	NO	Duplicate records
96	Mathematically defining and parametrically generating Traditional Chinese Private Gardens of the Suzhou Region and Style	NO	Duplicate records
97	Mathematically defining and parametrically generating Traditional Chinese Private Gardens of the Suzhou Region and Style	NO	Duplicate records
98	Parametrically Generating New Instances of Traditional Chinese Private Gardens that Replicate Selected Socio-Spatial and Aesthetic Properties	NO	Duplicate records
99	Parametrically Generating New Instances of Traditional Chinese Private Gardens that Replicate Selected Socio-Spatial and Aesthetic Properties	NO	Duplicate records
100	Spatial Syntax Analysis of the Evolution of the Water System and Garden Distribution Relationship in Suzhou: 13th-20th Centuries	NO	Duplicate records
101	Exploring the Distribution of Gardens in Suzhou City in the Qianlong Period through a Space Syntax Approach	NO	Duplicate records
102	Exploring the Distribution of Gardens in Suzhou City in the Qianlong Period through a Space Syntax Approach	NO	Duplicate records
103	Do configurational attributes matter in context of urban parks? Park pathway configurational attributes and senior walking	NO	Duplicate records
104	Do configurational attributes matter in context of urban parks? Park pathway configurational attributes and senior walking	NO	Duplicate records
105	Do configurational attributes matter in context of urban parks? Park pathway configurational attributes and senior walking	NO	Duplicate records
106	Can trail spatial attributes predict trail use level in urban forest park? An examination integrating GPS data and space syntax theory	NO	Duplicate records
107	Can trail spatial attributes predict trail use level in urban forest park? An examination integrating GPS data and space syntax theory	NO	Duplicate records
108	A Case Study Based on Space Syntax Theory: West Shu Garden of Qingxi, Dujiangyan Scenic Area	NO	Duplicate records
109	A Case Study Based on Space Syntax Theory: West Shu Garden of Qingxi, Dujiangyan Scenic Area	NO	Duplicate records
110	"Seeing" or "Being Seen": Research on the Sight Line Design in the Lion Grove Based on Visitor Temporal-Spatial Distribution and Space Syntax	NO	Duplicate records
111	"Seeing" or "Being Seen": Research on the Sight Line Design in the Lion Grove Based on Visitor Temporal-Spatial Distribution and Space Syntax	NO	Duplicate records
112	Combining GPS and space syntax analysis to improve understanding of visitor temporal-spatial behaviour: a case study of the Lion Grove in China	NO	Duplicate records
113	The Mathematics of Spatial Structure Evolution: Using Syntactical Data to Compare the Humble Administrator's Garden in the Sixteenth and Nineteenth Centuries	NO	Duplicate records
114	The Mathematics of Spatial Structure Evolution: Using Syntactical Data to Compare the Humble Administrator's Garden in the Sixteenth and Nineteenth Centuries	NO	Duplicate records
115	Analyzing Urban Parks for Older Adults' Accessibility in Summer Using Gradient Boosting Decision Trees: A Case Study from Tianjin, China	NO	Duplicate records
116	Analyzing Urban Parks for Older Adults' Accessibility in Summer Using Gradient Boosting Decision Trees: A Case Study from Tianjin, China	NO	Duplicate records
117	Does the Presence of Birdsongs Improve Perceived Levels of Mental Restoration from Park Use? Experiments on Parkways of Harbin Sun Island in China	NO	Duplicate records
118	Does the Presence of Birdsongs Improve Perceived Levels of Mental Restoration from Park Use? Experiments on Parkways of Harbin Sun Island in China	NO	Duplicate records
119	Atlas Río Mayo: Dynamic Territory and Inter-Scalar Waman-Samanas Landscape in the Peruvian Amazon. Case Study: Bajo Mayo Network Communities: Flores del Río Mayo, San Antonio del Río Mayo, Churuyacu del Río Mayo and Solo del Río Mayo. Lamas, San Martin	NO	Dissertation
120	Kuzey Makedonya'da Tetova şehrinin mekân yapilanmasindaki gizli ağThe Hidden Network in the Spatial Structure of the City of Tetova in North Macedonia	NO	Dissertation
121	A Historical Inquiry into the Failure of Downtown Eugene's Pedestrian Mall Strategy to Revitalize the Retail Core, 1971–2002	NO	Dissertation
122	The Utilization of Space Syntax Theories to Develop a Parametric and Generative Urban Design Program in Grasshopper	NO	Dissertation
123	Transitive Gestures: Everyday Structures at Play	NO	Dissertation
124	Environmental Psychology and Human Well-Being	NO	Book
125	Introduction: Rationale, Chapter Overviews, and Author Biographies	NO	Book
126	The Roman Veterans' Settlement at Moẓa c. AD 70–130	NO	Book
127	Sustainable Energy Transition for Cities	NO	Book

128	Chapter 26 - Urban design strategies and the smart city paradigm	NO	Book
129	Chapter 21 - Assessing the role of urban design in a rapidly urbanizing historical city and its contribution in restoring its urban ecology: the case of Varanasi, India	NO	Book
130	Chapter 9 - Impact of the natural and built environment on human health: A perspective from environmental psychology	NO	Book
131	Comprehensive Geographic Information Systems	NO	Book
132	Mapping Landscapes in Transformation	NO	Book
133	Political Landscapes of Capital Cities	NO	Book
134	Advances in Science, Technology and Innovation	NO	Book
135	Construction 4.0（61-149）	NO	Book
136	Construction 4.0（221-262）	NO	Book
137	Suburban Urbanities	NO	Book
138	Everyday Streets	NO	Book
139	Shaping Smart for Better Cities	NO	Book
140	The Role of Public Participation in Energy Transitions	NO	Book
141	Environmental Psychology and Human Well-Being	NO	Book
142	Suburban Urbanities	NO	Book
143	Western Mesoamerican Calendars and Writing Systems	NO	Book
144	Bending cylinders: A geometric syntax for zero-waste architecture	NO	Book
145	Intelligent Environments (Second Edition)	NO	Book
146	Mediterranean Green Buildings and Renewable Energy: Selected Papers from the World Renewable Energy Network's Med Green Forum	NO	Book
147	On Power in Architecture: From a Materialistic, Phenomenological, and Post-Structuralist Perspective	NO	Book
148	Handbook of Clinical Neurology	NO	Book
149	Suburban Urbanities	NO	Book
150	Citizenship, Democracy and Belonging in Suburban Britain	NO	Book
151	Citizenship, Democracy and Belonging in Suburban Britain	NO	Book
152	Historicizing Sunni Islam in the Ottoman Empire, c. 1450-c. 1750	NO	Book
153	Co-curating the City	NO	Book
154	Encyclopedia of the Human Brain (Second Edition)	NO	Book
155	New Mexico and the Pimería Alta	NO	Book
156	Artificial Intelligence in Urban Planning and Design	NO	Book
157	Beyond the Meme	NO	Book
158	Advances in Transportation and Health	NO	Book
159	Handbook of the Changing World Language Map	NO	Book
160	Springer Tracts in Civil Engineering	NO	Book
161	Suburban Urbanities	NO	Book
162	Venice Variations	NO	Book
163	Parliament Buildings	NO	Book
164	Encyclopedia of Renewable and Sustainable Materials	NO	Book
165	Suburban Urbanities	NO	Book
166	Encyclopedia of Libraries, Librarianship, and Information Science (First Edition)	NO	Book
167	Drawing Attention: Architecture in the Age of Social Media	NO	Book
168	Artificial Intelligence in Urban Planning and Design	NO	Book
169	Urban Form and Accessibility	NO	Book
170	Mapping Society	NO	Book
171	Disease, health and housing	NO	Book
172	Mapping Society（61-92）	NO	Book
173	Mapping Society（129-167）	NO	Book
174	Mapping Society（168-204）	NO	Book
175	Livable Streets 2.0	NO	Book
176	Suburban Urbanities	NO	Book
177	Cities Made of Boundaries（45-71）	NO	Book
178	Cities Made of Boundaries（203-257）	NO	Book
179	Cities Made of Boundaries（128-163）	NO	Book
180	Cities Made of Boundaries（258-295）	NO	Book
181	Cities Made of Boundaries（101-127）	NO	Book
182	Ecosystem and Territorial Resilience	NO	Book
183	Urban Rituals in Sacred Landscapes in Hellenistic Asia Minor	NO	Book
184	Alternative Pathways to Complexity	NO	Book

185	Urban Construction and Management Engineering IV	NO	Book
186	Comprehensive Geographic Information Systems	NO	Book
187	The Covert Life of Hospital Architecture	NO	Book
188	The Covert Life of Hospital Architecture	NO	Book
189	Postmodernity and Japanese Zen Buddhism: From a semiotics of places, the case of the Ryoan-ji dry garden	NO	Outside the eligible time period
190	Dis-orientation: Spatial abilities performance in London	NO	Outside the eligible time period
191	Wayfinding in the Barbican Centre: Effects of spatial layout and visual information on spatial problem solving process	NO	Outside the eligible time period
192	Social and spatial implications of community-based residential environments on crime in urban settings	NO	Outside the eligible time period
193	Spatial configuration, spatial cognition and spatial behaviour: The role of architectural intelligibility in shaping spatial experience	NO	Outside the eligible time period
194	The Chosen Path: Movement Pattern Analysis and Land-Use within Jasper National Park and the Central Canadian Rocky Mountains	NO	Outside the eligible time period
195	Park interpretations: An exploration of the spatial properties and urban performance of Regent's Park, London and Pedion Areos Park, Athens	NO	Outside the eligible time period
196	The Photograph as a Site of Writing	NO	Outside the eligible time period
197	Park syntax:: Measuring open space accessibility and "smart growth"	NO	Outside the eligible time period
198	Planning for Cycling	NO	Outside the eligible time period
199	Encyclopedia of Social Measurement	NO	Outside the eligible time period
200	International Encyclopedia of Housing and Home	NO	Outside the eligible time period
201	Developments in Sedimentology	NO	Outside the eligible time period
202	Developments in Sedimentology	NO	Outside the eligible time period
203	Encyclopedia of Social Measurement	NO	Outside the eligible time period
204	Rehumanizing Housing	NO	Outside the eligible time period
205	Intelligent Environments	NO	Outside the eligible time period
206	Philosophy of Technology and Engineering Sciences	NO	Outside the eligible time period
207	Smart Things	NO	Outside the eligible time period
208	Planning for Cycling	NO	Outside the eligible time period
209	International Encyclopedia of the Social & Behavioral Sciences	NO	Outside the eligible time period
210	THE LEARNING ENVIRONMENT VERSUS THE UNLEARNED DESIGN NORMS: THE EVIDENCE OF PURE SUBJUGATION OF SPACE PLANNING AND DESIGN STANDARD OF UNIVERSITY CAMPUS UNDER THE PFI PROCUREMENT METHOD	NO	Outside the eligible time period
211	The Creation of Complexity in Chinese Garden	NO	Outside the eligible time period
212	VISUALANALYSIS OF THE RELATION BETWEEN CONCENTRATED DISTRICTS OF KNOWLEDGE-BASED INDUSTRIES AND THIRD PLACES IN OSAKA CITY	NO	Outside the eligible time period
213	Whether Museum Spatial Experience Can Synthesize Its Exhibits in the Museum with Chinese Garden Configuration	NO	Outside the eligible time period
214	Organization Based on Space Syntax for Shared Parking in Central Business District	NO	Outside the eligible time period
215	HOW TO TRANSFORM CAR-DEPENDENT CITY INTO PEDESTRIAN CITY?	NO	Outside the eligible time period
216	Proceedings of the Annual International Conference on Mobile Computing and Networking, MOBICOM	NO	Outside the eligible time period
217	Disaster resilience and the social fabric of space	NO	Outside the eligible time period
218	Moorish architectural syntax and the reference to nature: A case study of Algiers	NO	Outside the eligible time period
219	The creation of complexity in chinese garden	NO	Outside the eligible time period
220	Optimality parsing and local cost functions in discontinuous grammar	NO	Outside the eligible time period
221	Visualanalysis of the relation between concentrated districts of Knowledge-based industries and third places in Osaka city	NO	Outside the eligible time period
222	Visual perception of traditional garden space in Suzhou, China: A case study with space syntax techniques	NO	Outside the eligible time period
223	Whether museum spatial experience can synthesize its exhibits in the museum with Chinese garden configuration	NO	Outside the eligible time period
224	Mediating the Design of a “Digital Park” in Vrilissia Athens	NO	Outside the eligible time period
225	Integrating the recreational spaces of Rangpur City Corporation, Bangladesh: A perspective from Space syntax	NO	Outside the eligible time period
226	Application of space syntax theory in study of urban parks and walking	NO	Outside the eligible time period
227	Active Design Guidelines: Promoting Physical Activity and Health in Design	NO	Outside the eligible time period
228	Advancing Computing, Communication, Control and Management	NO	Outside the eligible time period
229	3rd International Conference on Civil Engineering and Transportation, ICCET 2013	NO	Outside the eligible time period
230	The Learning Environment versus the Unlearned Design Norms: The Evidence of Pure Subjugation of Space Planning and Design Standards under the PFI Procurement	NO	Outside the eligible time period

	Method		
231	Procedia - Social and Behavioral Sciences	NO	Outside the eligible time period
232	Location based services—new challenges for planning and public administration?	NO	Outside the eligible time period
233	Raising Awareness on Mobility Costs for Households: A Lever for Changing Residential Choices and Improving Local Governance? Experimentation in the French Alpine Metropolitan Area	NO	Outside the eligible time period
234	Urban texture and space configuration: An essay on integrating socio-spatial analytical techniques	NO	Outside the eligible time period
235	The Ayia Varvara site project: A case of urban landscape design	NO	Outside the eligible time period
236	ANU Reporter - Vol.06, No.12	NO	Outside the eligible time period
237	Visual Access Opportunity Analysis of Neighborhood Park Users usingSpace Syntax Theory - Focused on Cheongju City<br>공간구문론을 이용한 근린공원 시각적 접근기회 분석 - 청주시를 중심으로	NO	Outside the eligible time period
238	Brain and Language: a Perspective from Sign Language	NO	Outside the eligible time period
239	Presence in blended spaces	NO	Outside the eligible time period
240	A virtual reality tool to measure shoppers' tenant mix preferences	NO	Outside the eligible time period
241	Indices of Pedestrian Behavior in Shopping Areas	NO	Outside the eligible time period
242	The geometry of crime	NO	Outside the eligible time period
243	The Isle of Dogs: Four development waves, five planning models, twelve plans, thirty-five years, and a renaissance … of sorts	NO	Outside the eligible time period
244	Mobility and Urban Planning Integration at City-regional Level in the Design of Urban Transport Interchanges (EC FP7 NODES Project–Task 3.2.1.)	NO	Outside the eligible time period
245	Understanding urban segregation: Issues of ethnicity, age and gender in Nicosia's public space	NO	Outside the eligible time period
246	Processing of Visually Presented Sentences in Mandarin and English Studied with fMRI	NO	Outside the eligible time period
247	Rational-design process and evaluation of street-lighting design for apartment complexes	NO	Outside the eligible time period
248	Analysis Theme Park Space Configuration Using Space Syntax<br>공간구문론을 활용한 주제공원의 공간 구성 분석	NO	Outside the eligible time period
249	Dissociation between linguistic and nonlinguistic gestural systems: A case for compositionality	NO	Outside the eligible time period
250	The neural correlates of highly iconic structures and topographic discourse in French Sign Language as observed in six hearing native signers	NO	Outside the eligible time period
251	Spatial distribution of urban pollution: civilizing urban traffic	NO	Outside the eligible time period
252	Planning for sustainable accessibility: Developing tools to aid discussion and decision-making	NO	Outside the eligible time period
253	Architecture, society and space—the high-density question re-examined	NO	Outside the eligible time period
253	Transport Interchange and Local Urban Environment Integration	NO	Outside the eligible time period
255	Prospect-Refuge theory and the textile-block houses of Frank Lloyd Wright: An analysis of spatio-visual characteristics using isovists	NO	Outside the eligible time period
256	Space syntax analysis of Central Inuit snow houses	NO	Outside the eligible time period
257	Safety becomes danger: dilemmas of drug-use in public space	NO	Outside the eligible time period
258	Spatio-symbolic oppositions in ritual and architecture	NO	Outside the eligible time period
259	Structural robustness of city road networks based on community	NO	Outside the eligible time period
260	Association between residential greenness and birth weight: Systematic review and meta-analysis	NO	Outside the eligible time period
261	New Urbanism Principles versus Urban Design Dimensions towards Behavior Performance Efficiency in Egyptian Neighbourhood Unit	NO	Outside the eligible time period
262	Development of an evaluation system for parks in neighborhood communities - Case study in Kitakyushu city, Japan	NO	Outside the eligible time period
263	Crime in a planned city: The case of Brasília	NO	Outside the eligible time period
264	Urban Brazil: past and future	NO	Outside the eligible time period
265	Energy retrofit to nearly zero and socio-oriented urban environments in the Mediterranean climate	NO	Outside the eligible time period
266	Health outcomes and quasi-supervised settings for street injecting drug use	NO	Outside the eligible time period
267	Urban layout, landscape features and pedestrian usage	NO	Outside the eligible time period
268	NOTES OF BRITISH ARCHITECTURAL CULTURE 1945-1965	NO	Outside the eligible time period
269	Creating sense of community: The role of public space	NO	Outside the eligible time period
270	The city and urban heat islands: A review of strategies to mitigate adverse effects	NO	Outside the eligible time period
271	Characterising the urban environment of UK cities and towns: A template for landscape planning	NO	Outside the eligible time period
272	Emerging relationships between design and use of urban park spaces	NO	Outside the eligible time period
273	Some qualitative aspects of the urban environment in developed countries	NO	Outside the eligible time period

274	Urban Waterfront Redevelopment in Greek Cities: A Framework for Redesigning Space	NO	Outside the eligible time period
275	Gap Analysis of Current Industrial Challenges and the State-of-the-Art in Pedestrian Modelling	NO	Outside the eligible time period
276	Mind the map! The impact of transit maps on path choice in public transit	NO	Outside the eligible time period
277	The space for culture and cognition	NO	Outside the eligible time period
278	A symmetry detector for map generalization and urban-space analysis	NO	Outside the eligible time period
279	Dense civilisations: the shape of cities in the 21st century	NO	Outside the eligible time period
280	Neighborhood physical activity opportunities for inner-city children and youth	NO	Outside the eligible time period
281	The Use of Spatial Analysis Techniques in Mapping Potential Natural Hazard Areas: A Case Study of Taiwan	NO	Outside the eligible time period
282	Configurational Analysis of Cheongju Wonheung-ee Eco-park Network Plan Using Space Syntax<br>공간구문론에 의한 청주 원흥이 생태공원 가로망배치의 적합성 분석	NO	Outside the eligible time period
283	Locational Decision of the Viewpoint Using GIS and Space Syntax<br>공간구문론과 GIS 를 이용한 조망점 위치결정	NO	Outside the eligible time period
284	A Study on the Development of Symbolic Places for Local Cultural Festival- Focused on the places for International Jazz Festival in Gapyeong<br>지역문화축제 활성화를 위한 상징공간 구축방안 연구 -가평 국제 재즈페스티벌 장소를 중심으로	NO	Outside the eligible time period
285	Quiet environment: Acoustics of vertical green wall systems of the Islamic urban form	NO	Outside the eligible time period
286	Developing a topological information extraction model for space syntax analysis	NO	Outside the eligible time period
287	The realization of the linguistic metafunctions in a sign language	NO	Outside the eligible time period
288	Investing in technology for tourism activities: Perspectives and challenges	NO	Outside the eligible time period
289	Differences in the spatial patterns of urban tourism in Vienna and Prague	NO	Outside the eligible time period
290	Razlike v prostorskih vzorcih mestnega turizma na Dunaju in v Pragi	NO	Outside the eligible time period
291	How retail entrepreneurs perceive the link between surveillance, feeling of security, and competitiveness of the retail store? A structural model approach	NO	Outside the eligible time period
292	Landscape Design and Cognitive Psychology	NO	Outside the eligible time period
293	Accessibility modelling: predicting the impact of planned transport infrastructure on accessibility patterns in Edinburgh, UK	NO	Outside the eligible time period
294	Fifth-, sixth-, and seventh- grade students' use of non-classroom spaces during recess: The case of three private schools in Izmir, Turkey	NO	Outside the eligible time period
295	Research on the Planning Characteristics for Low-rise Residential Areas in the Suburbs of Japan<br>일본도심근교의 저층주거지 계획특성 조사연구	NO	Outside the eligible time period
296	Linking the spatial syntax of cognitive maps to the spatial syntax of the environment	NO	Outside the eligible time period
297	Linking the spatial syntax of cognitive maps to the spatial syntax of the environment	NO	Outside the eligible time period
298	Spatial configuration, spatial cognition and spatial behaviour : the role of architectural intelligibility in shaping spatial experience	NO	Outside the eligible time period
299	Effects of access to public open spaces on walking: Is proximity enough?	NO	Outside the eligible time period
300	Using Space Syntax to Assess the Built Environment for Physical Activity: Applications to Research on Parks and Public Open Spaces	NO	Outside the eligible time period
301	Using Space Syntax to Assess the Built Environment for Physical Activity: Applications to Research on Parks and Public Open Spaces	NO	Outside the eligible time period
302	Towards contextually sensitive urban densification: Location-based softGIS knowledge revealing perceived residential environmental quality	NO	Outside the eligible time period
303	Analyzing yachting patterns in the Biesbosch National Park using GIS technology	NO	Outside the eligible time period
304	Analyzing yachting patterns in the Biesbosch National Park using GIS technology	NO	Outside the eligible time period
305	Analyzing yachting patterns in the Biesbosch National Park using GIS technology	NO	Outside the eligible time period
306	Healthy campus by open space design: Approaches and guidelines	NO	Outside the eligible time period
307	A psychological—spatial approach for architectural design and research	NO	Outside the eligible time period
308	Analysis of the Spatial Structure of Kazuyo Sejima & Ryue Nishizawa's House Designs<br>세지마 카즈요 및 니시자와 류에 주택의 공간구조분석 연구	NO	Outside the eligible time period
309	Space Syntactic Properties of the Street Network in Commercial District from the Perspective of Human Ecology - Focused on Euneungjeongi Culture Street in Daejeon, Korea<br>인간 생태학적 관점에서의 상업지구 내 가로망의 공간배열 특성 - 대전시 으능정이 문화거리를 중심으로	NO	Outside the eligible time period
310	Crime, fear of crime, environment, and mental health and wellbeing: Mapping review of theories and causal pathways	NO	Outside the eligible time period
311	Spatial interpolation of traffic counts based on origin–destination centrality	NO	Outside the eligible time period
312	Modelling of urban green space walkability: Eco-friendly walk score calculator	NO	Outside the eligible time period

313	Toward an integrated theory of spatial morphology and resilient urban systems	NO	Outside the eligible time period
314	Journal of Environmental Psychology	NO	Outside the eligible time period
315	A spatial analysis of the JBA headquarters in Splinter Cell: Double Agent	NO	Outside the eligible time period
316	Quantifying the defensiveness of defended sites on the Northwest Coast of North America	NO	Outside the eligible time period
317	The link between built environment, pedestrian activity and pedestrian–vehicle collision occurrence at signalized intersections	NO	Outside the eligible time period
318	Classification Analysis of the Physical Environment of Bicycle Road -Focused on Chang Won City, Kyung Nam Province, S. Korea<br>자전거 도로의 물리적 환경에 대한 등급화 연구 -창원시 사례를 중심으로	NO	Outside the eligible time period
319	PADI-Simul: an agent-based geosimulation software supporting the design of geographic spaces	NO	Outside the eligible time period
320	Logistic regression and cellular automata-based modelling of retail, commercial and residential development in the city of Ahmedabad, India	NO	Outside the eligible time period
321	The Correlation Between Spatial Characteristics and Utilization of City Parks: A Focus on Neighborhood Parks in Seoul, Korea	NO	Outside the eligible time period
322	The correlation between spatial characteristics and utilization of city parks: A focus on neighborhood parks in Seoul, Korea	NO	Outside the eligible time period
323	Night Time Social Behavior in Urban Outdoor Spaces of Shah Alam	NO	Outside the eligible time period
324	Urban spatial configuration and socio-economic residential differentiation: The case of Tel Aviv	NO	Outside the eligible time period
325	Reading urban spaces by the space-syntax method: A proposal for the South Haliç Region	NO	Outside the eligible time period
326	Site Selection of Wildlife Passage for Leopard Cat in Urban Area using Space Syntax<br>공간구문론을 이용한 도시 내 삵 이동통로 적지선정	NO	Outside the eligible time period
327	Development and application of the Pedestrian Environment Index (PEI)	NO	Outside the eligible time period
328	The Proposition of the Convergence of Individual and Community Resilience	NO	Outside the eligible time period
329	Evaluating accessibility to Bangkok Metro Systems using multi-dimensional criteria across user groups	NO	Outside the eligible time period
330	The Crime Ecology: Ambient Temperature vs. Spatial Setting of Crime (Burglary)	NO	Outside the eligible time period
331	Built environment configuration and change in body mass index: The Caerphilly Prospective Study (CaPS)	NO	Outside the eligible time period
332	Maintaining style of garden designs by using graph-based constraints	NO	Outside the eligible time period
333	My neighbourhood: Studying perceptions of urban space and neighbourhood with moblogging	NO	Outside the eligible time period
334	Spatial modeling of bicycle activity at signalized intersections	NO	Outside the eligible time period
335	Classification and feature extraction of criminal occurrence points using CAEP with transductive clustering	NO	Outside the eligible time period
336	The broken middle: The space of the London riots	NO	Outside the eligible time period
337	Innovation spaces: Workspace planning and innovation in U.S. university research centers	NO	Outside the eligible time period
338	Visual Presentation of Mental Images in Urban Design Education:Cognitive Maps	NO	Outside the eligible time period
339	An extensible simulation environment and movement metrics for testing walking behavior in agent-based models	NO	Outside the eligible time period
340	Industrial land use efficiency under government intervention: Evidence from Hangzhou, China	NO	Outside the eligible time period
341	Environmental Sustainability of Newly Developed City Squares in Historic Cities: Case Study of Isfahan-Iran	NO	Outside the eligible time period
342	Comparative floorplan-analysis in programming and architectural design	NO	Outside the eligible time period
343	Conceptions of Space in the Evolution of a Gridded and an Organic Town: Bloemfontein and Kimberley, South Africa	NO	Outside the eligible time period
344	The spatial syntax of urban segregation	NO	Outside the eligible time period
345	Past and current trends in sign language research	NO	Outside the eligible time period
346	On(not) choosing between mobility and visibility<br>Crossing sexual and national borders in Israel/Palestine	NO	Outside the eligible time period
347	Older people's navigation of urban areas as pedestrians: Measuring quality of the built environment using oral narratives and virtual routes	NO	Outside the eligible time period
348	Street centrality and land use intensity in Baton Rouge, Louisiana	NO	Outside the eligible time period
349	A mixed integer programming formulation and solution for traffic analysis zone delineation considering zone amount decision	NO	Outside the eligible time period
350	Pricing accessibility: Urban morphology, design and missing markets	NO	Outside the eligible time period
351	The structure of CAD and the structure of form	NO	Outside the eligible time period
352	Brasília after Brasília	NO	Outside the eligible time period
353	A Study on the Correlations between Spatial Configuration and Neighborhood	NO	Outside the eligible time period

	Environment of Gangnam-dong, Jinju City - Through Comparison Analysis with Chilam-dong, Jinju City<br>진주시 강남동의 공간구조와 주거 Â·근린상업 환경의 상관성 연구 - 진주시 칠암동과의 비교 분석을 통하여		
354	Spatial Structure Analysis of Private Arboretum using Space Syntax Method : Focused on Jade Garden Arboretum<br>Space Syntax 를 활용한 사립수목원 공간구조분석 : 제이드가든 수목원을 중심으로	NO	Outside the eligible time period
355	Impacts of land reclamation on the landscape of Lake Biwa, Japan	NO	Outside the eligible time period
356	The Application of Modern Space Syntax Theory in Residential Gardens	NO	Outside the eligible time period
357	A study on Strategy of Interpretation towards Classical Garden by Chinese Modern Architecture	NO	Outside the eligible time period
358	Influences of building design and site design on physical activity: Research and intervention opportunities	NO	Outside the eligible time period
359	How to Treat “BA”construction in Machine Translation<br>기계번역에서의 把자문 처리 기제	NO	Outside the eligible time period
360	Study on “BEI”construction in Machine Translatio<br>기계번역에서의 ‘被’자문 처리 기제	NO	Outside the eligible time period
361	Open space quality in deprived urban areas: user perspective and use pattern	NO	Conference
362	COMPUTATIONAL ANALYSIS AND GENERATION OF TRADITIONAL CHINESE PRIVATE GARDENS THROUGH SPACE SYNTAX AND PARAMETRIC DESIGN	NO	Conference
363	Picturesque Garden Design in Early 18th Century The Stourhead	NO	Conference
364	Accessibility and Polarities of Pedestrian Network in University Campuses. A Space Syntax Application	NO	Conference
365	Spatial Form Analysis of Existing Industrial Buildings Renovation Based on Space Syntax	NO	Conference
366	Decoding and Predicting the Attributes of Urban Public Spaces with Soft Computing Models and Space Syntax Approaches	NO	Conference
367	Wayfinding in traditional Chinese private gardens: a spatial analysis of the Yuyuan garden	NO	Conference
368	Suitability analyses between exercise patterns of morning exercise and green space characteristics: A case study of Zhaolin park, China	NO	Conference
369	5th International Conference Planning in the Era of Uncertainty, ICPEU 2021	NO	Conference
370	2023 International Conference on Architectural Research and Design (ARDC) in conjunction with CIVPLAN'S International Conference	NO	Conference
371	The pursuit of a sustainable and accessible mobility on university campuses	NO	Conference
372	Disciplined informality: Assembling un-programmed spatial practices in three public libraries in Medellín	NO	Conference
373	Biodiversity Potential in Brazilian Medium-sized Cities through Human Access to Nature	NO	Conference
374	2014 World Cup: A motivation for accessibility improvement in Porto Alegre, Brazil	NO	Conference
375	A study on the coffeeshop space in Vinh Long City, Vietnam	NO	Conference
376	Space after dark: Measuring the impact of public lighting at night on visibility, movement, and spatial configuration in urban parks	NO	Conference
377	Lessons from the Distribution Pattern of Urban Parks and Factors that Contribute to Control COVID-19 Outbreak in Neighborhood Design	NO	Conference
378	URBAN FABRICS OF HOUSING FORMS AND ITS IMPACT ON HUMAN BEHAVIOR	NO	Conference
379	Computational analysis and generation of traditional Chinese private gardens through space syntax and parametric design	NO	Conference
380	Space Syntax and Disability: Can Space Syntax Predict Users with Disabilities’ Movement? A case study from Algiers’ historical city of Casbah	NO	Conference
381	Distances, accessibilities and attractiveness: Urban form correlates of willingness to pay for dwellings examined by space syntax based measurements in GIS	NO	Conference
382	Making the megacity for everyone: Towards inclusiveness and vitality for Shenzhen's urban periphery	NO	Conference
383	PEDESTRIAN ACCESSIBILITY ASSESSMENT USING SPATIAL AND NETWORK ANALYSIS: A CASE OF SOFIA CITY	NO	Conference
384	Formal adaptability: A discussion of morphological changes and their impact on density in low-rise mass housing	NO	Conference
385	Evaluating the impacts of an urban design project: Multi-phase Analyses of Taksim Square and Gezi Park, Istanbul	NO	Conference
386	Research on the Distribution Characteristics of Public Charging Piles Supported by	NO	Conference

	Multi-source Data - - Taking Guangzhou City as an Example		
387	Spatial and temporal communication of burglary risk	NO	Conference
388	Accessibility and Polarities of Pedestrian Network in University Campuses. A Space Syntax Application	NO	Conference
389	Applying space syntax analysis in the design process of a single-family house conversion aiming at sustainability and safety	NO	Conference
390	Wandering, observing and mapping. Understanding the "geography of accessibility" in the cultural landscape of sintra	NO	Conference
391	The Apple story: Spatial, functional and cultural parameters in branded architecture	NO	Conference
392	Investigating the Spatial Suitability of the Location of Urban Services Using Space Syntax Theory	NO	Conference
393	Sketch-a-park Study of the usability of diagrams for participatory sketching	NO	Conference
394	The socio-spatial relations of the accessibility of parks in chicago	NO	Conference
395	Proposing urban park coverage area using spatial configuration approach towards the more sustainable movement in Malang City	NO	Conference
396	Using space syntax as a tool for architectural design	NO	Conference
397	Space syntax. Mathematical analysis of traditional Chinese private gardens planning structures	NO	Conference
398	Vitality and urban voids in goiânia (brazil): The south sector case	NO	Conference
399	How does a child act in a theme park? Searching the role of space syntax in a child's cognitive schema	NO	Conference
400	Patient Control Mechanisms in the 19th century Asylums of England: A Comparative Space Syntax Analysis of Asylums Based on Patient Holding Capacity	NO	Conference
401	Exploring the levels of availability and access to open/green space and health outcomes The case of Camden, London	NO	Conference
402	Spatial morphology of rebel cities: The cases of Madrid, Merida and Istanbul	NO	Conference
403	Spatial configuration shapes student social and informal learning activities in educational complexes	NO	Conference
404	Cultural impacts on traditional Chinese garden design: A configurational comparison between traditional Chinese imperial and private gardens using space syntax	NO	Conference
405	Creating and connecting new development areas: an emphasis on cyclists and pedestrians An examination of Bailrigg Garden village	NO	Conference
406	A quantitative study of the layout of Wangshi Yuan by means of Space Syntax analysis	NO	Conference
407	Decoding and Predicting the Attributes of Urban Public Spaces with Soft Computing Models and Space Syntax Approaches	NO	Conference
408	Trail configurational attributes and visitors' spatial distribution in natural recreation area	NO	Conference
409	Evaluation and Analysis of Subway Accessibility Based on Spatial Syntax: A Case Study of Xi'an	NO	Conference
410	Suitability Analyses between Exercise Patterns of Morning Exercise and Green Space Characteristics: A Case Study of Zhaolin Park, China	NO	Conference
411	Annotated Bibliography on Land Value Taxation and Value Capture (With a little bit on the Henry George Theorem) 2007-2013	NO	Conference
412	Understanding Urban Economies, Land Use, and Social Dynamics in the City: Big Data and Measurement	NO	Conference
413	Digital Connector: integrating hard and soft models to solve urban challenges	NO	Abstract and keywords
414	Form Leads to Function: Multi-scale modelling of Lymph Node Architecture in Response to Vaccine Adjuvants	NO	Abstract and keywords
415	Erratum regarding missing Declaration of Competing Interest statements in previously published articles (Frontiers of Architectural Research (2019) 8(2) (238–260), (S209526351930010X), (10.1016/j.foar.2019.03.001))	NO	Abstract and keywords
416	Erratum regarding missing Declaration of Competing Interest statements in previously published articles	NO	Abstract and keywords
417	BHAAAS International Conference on Sustainable Development, ICSD 2022, held as part of the 13th Days of Bosnian-Herzegovinian American Academy of Arts and Sciences, 2022	NO	Abstract and keywords
418	Statement of retraction: Supply–demand matching and coupling coordination characteristics of urban park green space from a health perspective (Intelligent Buildings International, (2023), (1-15), 10.1080/17508975.2023.2166895)	NO	Abstract and keywords
419	Editorial Board	NO	Abstract and keywords
420	Open Space Quality in Deprived Urban Areas: User Perspective and Use Pattern	NO	Abstract and keywords
421	SPACE SYNTAX AS A TOOL TO MEASURE SAFETY IN SMALL URBAN PARKS - A CASE STUDY OF ROD EL FARAG PARK IN CAIRO, EGYPT	NO	Abstract and keywords
422	The role of ICTs in creating the new social public place of the digital era	NO	Abstract and keywords
423	A hybrid simulation-assignment modeling framework for crowd dynamics in large-	NO	Abstract and keywords

	scale pedestrian facilities		
424	The Influence of Permeability on Social Cohesion: Is it Good or Bad?	NO	Abstract and keywords
425	Urban design assessment tools: a model for exploring atmospheres and situations	NO	Abstract and keywords
426	Urban form and air pollution: Clustering patterns of urban form factors related to particulate matter in Seoul, Korea	NO	Abstract and keywords
427	Measuring pedestrians' movement and building a visual-based attractiveness map of public spaces using smartphones	NO	Abstract and keywords
428	Diving for the spatio-functional qualities of exclusivity at The Pearl-Qatar	NO	Abstract and keywords
429	Modelling cyclists' route choice using Strava and OSMnx: A case study of the City of Glasgow	NO	Abstract and keywords
430	Making religious buildings more accessible<br>The case of mosques in Abu Dhabi's and Dubai's neighborhoods	NO	Abstract and keywords
431	From local to global: Uniting neighborhood planning units for more efficient walks	NO	Abstract and keywords
432	Planning in the age of pandemics: Renewing suburban design	NO	Abstract and keywords
433	Shade maps for prioritizing municipal microclimatic action in hot climates: Learning from Tel Aviv-Yafo	NO	Abstract and keywords
434	Common architectural characteristics of traditional courtyard houses in Erbil city	NO	Abstract and keywords
435	How do urban designs shape residential neighbourhood walkability? Evidence from metropolitan Lagos	NO	Abstract and keywords
436	Improving the Quality of Public Open Spaces in Hama, Syria: An Investigation through the Social Spatial Approach	NO	Abstract and keywords
437	Relationship between physical elements and density of use of public spaces in Sana'a City	NO	Abstract and keywords
438	Natural surveillance characteristics of building openings and relationship to residential burglary	NO	Abstract and keywords
439	A stated preference model to value reductions in community severance caused by roads	NO	Abstract and keywords
440	Community Severance: Where Is It Found and at What Cost?	NO	Abstract and keywords
441	Tempo-spatial analysis of pedestrian movement in the built environment based on crowdsourced big data	NO	Abstract and keywords
442	Re-examination of the standards for transit oriented development influence zones in India	NO	Abstract and keywords
443	Barriers to accessibility of urban roads by persons with disabilities: A review of the literature	NO	Abstract and keywords
444	The role of urban furniture in promoting gender equality and static social activities in public spaces	NO	Abstract and keywords
445	Exploring the role of configurational accessibility of alleyways on facilitating wayfinding transportation within the organic street network systems	NO	Abstract and keywords
446	The influence of social interactions on the behavioral patterns of the people in urban spaces (case study: The pedestrian zone of Rasht Municipality Square, Iran)	NO	Abstract and keywords
447	Predicting the Unpredictable-A case Analysis of Kozhikode City	NO	Abstract and keywords
448	Probabilistic modeling framework for multisource sound mapping	NO	Abstract and keywords
449	A high resolution agent-based model to support walk-bicycle infrastructure investment decisions: A case study with New York City	NO	Abstract and keywords
450	Modelling characteristics of the urban form to support water systems planning	NO	Abstract and keywords
451	Understanding spatial growth of the old city of Nanjing during 1850–2020 based on historical maps and Landsat data	NO	Abstract and keywords
451	Estimating Incoming Cross-border Trips Through Land Use data Resources – A Case of Karachi City	NO	Abstract and keywords
453	Urban structure to determine equitable city growth for spatial justice: A case study of Chía-Bogotá, Colombia	NO	Abstract and keywords
454	Low-income housing layouts under socio-architectural complexities: A parametric study for sustainable slum rehabilitation	NO	Abstract and keywords
455	Detecting the physical aspects of local identity using a hybrid qualitative and quantitative approach: The case of Souk Al-Khawajat district	NO	Abstract and keywords
456	A New Approach to Understand Modal and Pedestrians Route in Portugal	NO	Abstract and keywords
457	Global microscale walkability ratings and rankings: A novel composite indicator for 59 European city centres	NO	Abstract and keywords
458	Professional patios, emotional studios: Locating social ties in European art residences	NO	Abstract and keywords
459	Social retrofitting design through occupancy pattern in Tanta University, Egypt	NO	Abstract and keywords
460	On the impact of safety requirements, energy prices and investment costs in street lighting refurbishment design	NO	Abstract and keywords
461	Urban landscape structure anatomy: Structure patterns and typology identification in the space-time of Setif City, Algeria	NO	Abstract and keywords
462	Fragmentation and Diversity of the "Valdaisky" National Park Forests	NO	Abstract and keywords

462		NO	Abstract and keywords
463	From smart to empathic cities	NO	Abstract and keywords
464	Urban structure, spatial equilibrium, and social inequality at Ancient Teotihuacan	NO	Abstract and keywords
465	OSMnx: New methods for acquiring, constructing, analyzing, and visualizing complex street networks	NO	Abstract and keywords
466	Modelling of urban activity centers in Tokyo from alternative indicators of urban activity	NO	Abstract and keywords
467	Patterns of temporal and spatial variability of parking in a large City in the context of road network configuration – The case of Łódź, Poland	NO	Abstract and keywords
468	Urban Connectivity: Elements for an Identification of Bir El Bey's Preferential Landscapes	NO	Abstract and keywords
469	Pedestrian crossings: Design recommendations do not reflect users' experiences in a car-dominated environment in Auckland, New Zealand	NO	Abstract and keywords
470	Explore the application of reinforced learning to support decision making during the design phase in the construction industry	NO	Abstract and keywords
471	Effects of COVID-19 pandemic on spatial preferences and usage habits of users in shopping malls and its relation with circulation layout	NO	Abstract and keywords
472	Analysis of mobility on universities campuses in metropolises of emerging countries through the combination of inductive reasoning and monographic procedure methods	NO	Abstract and keywords
473	The pursuit of a sustainable and accessible mobility on university campuses	NO	Abstract and keywords
474	Sensing multiple semantics of urban space from crowdsourcing positioning data	NO	Abstract and keywords
475	Typological diversity and morphological continuity in the modern residential fabric: The case of Ankara, Turkey	NO	Abstract and keywords
476	Green walking networks for climate change adaptation	NO	Abstract and keywords
478	London's local high streets: The problems, potential and complexities of mixed street corridors	NO	Abstract and keywords
479	Street appeal: The value of street improvements	NO	Abstract and keywords
480	Measuring urban forms from inter-building distances: Combining MST graphs with a Local Index of Spatial Association	NO	Abstract and keywords
481	Planning for nodes, places, and people in Flanders and Brussels<br>An empirical railway station assessment tool for strategic decision-making	NO	Abstract and keywords
482	Fuzzy modeling for route characteristic	NO	Abstract and keywords
483	Determining thresholds for spatial urban design and transport features that support walking to create healthy and sustainable cities: findings from the IPEN Adult study	NO	Abstract and keywords
484	Urban and architectural spatial changes based on technology-adapted users: A literature review	NO	Abstract and keywords
485	Impacts of connections to the outside on underground space occupants' psychophysiological health: A virtual reality-based experimental approach	NO	Abstract and keywords
486	Cycling injury risk in Britain: A case-crossover study of infrastructural and route environment correlates	NO	Abstract and keywords
487	Modeling an Indian megalopolis– A case study on adapting SLEUTH urban growth model	NO	Abstract and keywords
488	An agent-based model of public space use	NO	Abstract and keywords
489	Spatial disparities in urban park accessibility: Integrating real-time traffic data and housing prices in Ningbo, China	NO	Abstract and keywords
490	Bicycle parking security and built environments	NO	Abstract and keywords
491	A systematic review of alternative protocols for evaluating non-spatial dimensions of urban parks	NO	Abstract and keywords
492	Classification of urban morphology with deep learning: Application on urban vitality	NO	Abstract and keywords
493	Spatial scaling effects analysis of existing historical streets in Chinese cities based on the Ping Ge type map—In case study of Quanzhou East–West Street	NO	Abstract and keywords
494	Industrial information integration—A literature review 2006–2015	NO	Abstract and keywords
495	A study on street walkability for older adults with different mobility abilities combining street view image recognition and deep learning - The case of Chengxianjie Community in Nanjing (China)	NO	Abstract and keywords
496	Construction of a public space network connectivity and vitality optimization system in historical districts of southwestern China: Guiyang case study	NO	Abstract and keywords
497	Flow-based unit is better: exploring factors affecting mid-term OD demand of station-based one-way electric carsharing	NO	Abstract and keywords
498	From service capacity to spatial equity: Exploring a multi-stage decision-making approach for optimizing elderly-care facility distribution in the city centre of Tianjin, China	NO	Abstract and keywords
499	City gate as key towards sustainable urban redevelopment: A case study of ancient Gungnae City within the modern city of Ji'an	NO	Abstract and keywords
500	Metric or topological proximity? The associations among proximity to parks, the	NO	Abstract and keywords

	frequency of residents' visits to parks, and perceived stress		
501	Estimating outdoor advertising media visibility with voxel-based approach	NO	Abstract and keywords
502	Survival characteristics of food and beverage businesses in a gentrified commercial area—A case study in Seoul, Korea	NO	Abstract and keywords
503	Effect of street network design on traffic congestion and traffic safety	NO	Abstract and keywords
504	Using spatial network analysis to model pedal cycle flows, risk and mode choice	NO	Abstract and keywords
505	sDNA: 3-d spatial network analysis for GIS, CAD, Command Line & Python	NO	Abstract and keywords
506	A new type of cities for liveable futures. Isobenefit Urbanism morphogenesis	NO	Abstract and keywords
507	Mathematize urbes by humanizing them. Cities as isobenefit landscapes: psycho-economical distances and personal isobenefit lines	NO	Abstract and keywords
508	Location choice with longitudinal WiFi data	NO	Abstract and keywords
510	Factors affecting beach walkability- Tourists' perception study at selected beaches of West Bengal, India	NO	Abstract and keywords
511	'Visual excitation' in Richard Neutra's residential architecture: An analysis using weighted graphs and centrality measures	NO	Abstract and keywords
512	Street and activity centre characteristics associated with the use of different transport modes	NO	Abstract and keywords
513	Mat-hybrid housing: Two case studies in Terni and London	NO	Abstract and keywords
514	Performance-driven design methodology for habitation shell design in extreme conditions on Mars	NO	Abstract and keywords
515	Machine learning-aided generative design methodology for a Martian regolith habitation shell	NO	Abstract and keywords
516	An evacuation model validation data-set for high-rise construction sites	NO	Abstract and keywords
517	A multi-scale user-friendliness evaluation approach on cycling network utilizing multi-source data	NO	Abstract and keywords
518	Perceived and objective measures of neighborhood environment: Association with active commuting to school by socioeconomic status in Brazilian adolescents	NO	Abstract and keywords
519	Stress and streets: How the network structure of streets is associated with stress-related brain activation	NO	Abstract and keywords
520	Effects of road network characteristics on bicycle safety: A multivariate Poisson-lognormal model	NO	Abstract and keywords
521	Quantifying the heterogeneity impact of risk factors on regional bicycle crash frequency: A hybrid approach of clustering and random parameter model	NO	Abstract and keywords
522	Built environment and urban cruise tourists' mobility	NO	Abstract and keywords
523	Night and day at the beach: Relating social life to location and infrastructure in a Brazilian city	NO	Abstract and keywords
524	Multi-dimensional urban segregation in João Pessoa, a coastal Brazilian northeastern city	NO	Abstract and keywords
525	Assessing impacts of objective features and subjective perceptions of street environment on running amount: A case study of Boston	NO	Abstract and keywords
526	Research progress and prospects for constructing ecological security pattern based on ecological network	NO	Abstract and keywords
527	Does every public open space (POS) contribute to sustainable city development? An assessment of inefficient POS in Beijing	NO	Abstract and keywords
528	Automatic identification and feature recognition of the metro-led underground space in China based on point of interest data	NO	Abstract and keywords
529	Quantitative assessment method on urban vitality of metro-led underground space based on multi-source data: A case study of Shanghai Inner Ring area	NO	Abstract and keywords
530	Pre-evacuation path planning in chemicals-concentrated areas with risk assessment and individual character analysis	NO	Duplicate records
531	Pre-evacuation path planning in chemicals-concentrated areas with risk assessment and individual character analysis	NO	Duplicate records
532	Pre-evacuation path planning in chemicals-concentrated areas with risk assessment and individual character analysis	NO	Abstract and keywords
533	Towards a morphogenesis of informal settlements	NO	Abstract and keywords
534	How neoliberal globalization processes are transforming Kyiv's nodal areas	NO	Abstract and keywords
535	Kako neoliberalni globalizacijski procesi preobražajo vozlišča v Kijevu	NO	Abstract and keywords
536	Identifying critical nodes in metro network considering topological potential: A case study in Shenzhen city—China	NO	Abstract and keywords
537	Women's perceived safety in public places and public transport: A narrative review of contributing factors and measurement methods	NO	Abstract and keywords
538	Joint associations and pathways from greenspace, traffic-related air pollution, and noise to poor self-rated general health: A population-based study in Sofia, Bulgaria	NO	Abstract and keywords
539	Professionals' perceptions for designing vibrant public spaces: Theory and praxis	NO	Abstract and keywords
540	Monitoring the transformation in New Cairo's urban vitality and the accompanying	NO	Abstract and keywords

	social and economic phenomena		
541	What makes livable cities of today alike? Revisiting the criterion of singularity through two case studies	NO	Abstract and keywords
542	Data-driven urban management: Mapping the landscape	NO	Abstract and keywords
543	Capturing the value of green space in urban parks in a sustainable urban planning and design context<br>pros and cons of hedonic pricing	NO	Abstract and keywords
544	Evaluate user satisfaction for urban design of railway station areas: An assessment framework using agent-based simulation	NO	Abstract and keywords
545	Urban carrying capacity assessment framework for mega mall development. A case study of Tehran's 22 municipal districts	NO	Abstract and keywords
546	Weather conditions and ski resorts' vitality: Linear and non-linear effects	NO	Abstract and keywords
547	Spatial heterogeneity modeling of city prosperity using GWt-test: The case study of Tehran	NO	Abstract and keywords
548	The Role of Urban Morphology Design on Enhancing Physical Activity and Public Health	NO	Abstract and keywords
549	Proximity to high streets, social isolation and social support in British adolescents: A longitudinal analysis of sociospatial influences on social connectedness using geospatial data	NO	Abstract and keywords
550	Perception of urban subdivisions in pedestrian movement simulation	NO	Abstract and keywords
551	A computational approach to 'The Image of the City'	NO	Abstract and keywords
552	Built environment attributes and their influence on walkability	NO	Abstract and keywords
553	Detroit's lines of desire: Footpaths and vacant land in the Motor City	NO	Abstract and keywords
554	Beyond the built-up form/mobility relationship: Spatial affordance and lifestyles	NO	Abstract and keywords
555	Crafting a jogging-friendly city: Harnessing big data to evaluate the runnability of urban streets	NO	Abstract and keywords
556	Do urban park spatial features influence public emotional responses during jogging? Evidence from social media data	NO	Abstract and keywords
557	Accessibility and satisfaction with daily commute: Does subjective perception in accordance with objective measurement?	NO	Abstract and keywords
558	Visual comfort impact assessment for walking spaces of urban historic district in China based on semantic segmentation algorithm	NO	Abstract and keywords
559	Sustainability-oriented configurational analysis of the street network of China's superblocks: Beyond Marshall's model	NO	Abstract and keywords
560	Developing an indicative spatial accessibility analysis tool for urban public transportation system	NO	Abstract and keywords
561	The role of urban configuration during disasters. A scenario-based methodology for the post-earthquake emergency management of Italian historic centres	NO	Abstract and keywords
562	A computational perspective on the dynamics of early architecture	NO	Abstract and keywords
563	The size of cities: A synthesis of multi-disciplinary perspectives on the global megalopolis	NO	Abstract and keywords
564	Morphological analysis of historical lanscapes based on cultural DNA approach	NO	Abstract and keywords
565	Morphological analysis of historical landscapes based on cultural DNA approach	NO	Abstract and keywords
566	Walkability for children in Bologna: Beyond the 15-minute city framework	NO	Abstract and keywords
567	(Ad)dressing belonging in a contested space: Embodied spatial practices of Palestinian and Israeli women in Jerusalem	NO	Abstract and keywords
568	Pedestrian safety perception and urban street settings: a comment	NO	Abstract and keywords
569	London taxi drivers exploit neighbourhood boundaries for hierarchical route planning	NO	Abstract and keywords
570	A crime risk-based approach for urban planning. A methodological proposal	NO	Abstract and keywords
571	Construction and evaluation of ecological networks among natural protected areas based on "quality-structure–function": A case study of the Qinghai-Tibet area	NO	Abstract and keywords
572	A comprehensive review of emergency department simulation applications for normal and disaster conditions	NO	Abstract and keywords
573	The Impacts of Urban Morphology on Urban Heat Islands in Housing Areas: The Case of Erzurum, Turkey	NO	Abstract and keywords
574	The Evaluation of the Perceptibility and Accessibility: The Case of Gaziantep	NO	Abstract and keywords
575	Assessing the ecological performance of French territories using a spatially-nested approach	NO	Abstract and keywords
576	Campus score: Measuring university campus qualities	NO	Abstract and keywords
577	Multi-objective unequal area pod-structured healthcare facility layout problem with daylight requirements	NO	Abstract and keywords
578	Promoting climate-driven forest migration through large-scale urban afforestation	NO	Abstract and keywords
579	Integrated effects of urban morphology on birdsong loudness and visibility of green areas	NO	Abstract and keywords

580	Walking as soft mobility: A multi-criteria GIS-based approach for prioritizing tourist routes	NO	Abstract and keywords
581	A spatial design network analysis of street networks and the locations of leisure entertainment activities: A case study of Wuhan, China	NO	Abstract and keywords
582	Children's school commuting in the Netherlands: Does it matter how urban form is incorporated in mode choice models?	NO	Abstract and keywords
583	Natural and built environmental exposures on children's active school travel: A Dutch global positioning system-based cross-sectional study	NO	Abstract and keywords
584	Resilience in Latin American Cities: Behaviour vs. Space quality in the Riverbanks of the Tomebamba River	NO	Abstract and keywords
585	Quantifying the usage of small public spaces using deep convolutional neural network	NO	Abstract and keywords
586	Impacts of corridor design: An investigation on occupant perception of corridor forms in elderly facilities	NO	Abstract and keywords
587	A spatiotemporal intelligent framework and experimental platform for urban digital twins	NO	Abstract and keywords
588	Green-gray imbalance: Rapid urbanization reduces the probability of green space exposure in early 21st century China	NO	Abstract and keywords
589	Integer programming for urban design	NO	Abstract and keywords
590	A city is not a tree: a multi-city study on street network and urban life	NO	Abstract and keywords
591	The image of the City on social media: A comparative study using “Big Data” and “Small Data” methods in the Tri-City Region in Poland	NO	Abstract and keywords
592	A binomial distribution model for describing pedestrian-vehicle crashes in urban areas	NO	Abstract and keywords
593	Advancing a novel large-scale assessment integrating ecosystem service flows and real human needs: A comparison between China and the United States	NO	Abstract and keywords
594	Doctoral research on architecture in Nigeria: Exploring domains, extending boundaries	NO	Abstract and keywords
595	Morphology of brothels: An investigation of spatial censorship and stigmatization - the case of Adana, Turkey	NO	Abstract and keywords
596	Flood disaster evacuation route choice in Indonesian urban riverbank kampong: Exploring the role of individual characteristics, path risk elements, and path network configuration	NO	Abstract and keywords
597	The Pedestrian Network Concept: A Systematic Literature Review	NO	Abstract and keywords
598	Urban Green Accessibility Index: A Measure of Pedestrian-Centered Accessibility to Every Green Point in an Urban Area	NO	Abstract and keywords
599	A Study on Pedestrian Satisfaction Among the Mobility Disadvantaged Persons According to Urban Form	NO	Abstract and keywords
600	Generative AI in architectural design: Application, data, and evaluation methods	NO	Abstract and keywords
601	Measuring Geometric Properties of Urban Blocks in Baghdad: A Comparative Approach	NO	Abstract and keywords
602	Travelers’ Route Choice: Comparing Relative Importance of Metric, Topological and Geometric Distance	NO	Abstract and keywords
603	Application for developing countries: Estimating trip attraction in urban zones based on centrality	NO	Abstract and keywords
604	Assessment of impact of bottlenecks on evacuation in subway stations using network analysis	NO	Abstract and keywords
605	Characteristics of spatial configurations in Pyongyang, North Korea	NO	Abstract and keywords
606	An analysis on the characteristics of factors for the healing environment in healthcare facilities - focused on the analysis of dankook university hospital and dental hospital in dankook university cheonan campus	NO	Abstract and keywords
607	Mapping urban public spaces based on the Nolli map method	NO	Abstract and keywords
608	Non-linear effects of street patterns and land use on the bike-share usage	NO	Abstract and keywords
609	A design-support framework to assess urban green spaces for human wellbeing	NO	Abstract and keywords
610	Resulting of pedigree and topology of centripetal spatial schema in Chinese traditional cities	NO	Abstract and keywords
611	A new approach to detecting and designing living structure of urban environments	NO	Abstract and keywords
612	Sustainable urban road planning under the digital twin-MCDM-GIS framework considering multidisciplinary factors	NO	Abstract and keywords
613	Generative urban design: A systematic review on problem formulation, design generation, and decision-making	NO	Abstract and keywords
614	Systematic framework for sustainable urban road alignment planning	NO	Abstract and keywords
615	Gaps and requirements for applying automatic architectural design to building renovation	NO	Abstract and keywords
616	Street vitality and built environment features: A data-informed approach from fourteen Chinese cities	NO	Abstract and keywords
617	Reconstructing urban spatial blind areas in child trafficking: Insights from the internet video surveillance data	NO	Abstract and keywords

618	Nonlinear public transit accessibility effects on housing prices: Heterogeneity across price segments	NO	Abstract and keywords
619	The relationship between walkability and neighborhood social environment: The importance of physical and perceived walkability	NO	Abstract and keywords
620	Tourism flows in large-scale destination systems	NO	Abstract and keywords
621	The effects of spatial accessibility and centrality to land use on walking in Seoul, Korea	NO	Abstract and keywords
622	Measuring the effects of street network configurations on walking in Seoul, Korea	NO	Abstract and keywords
623	The S + 5Ds: Spatial access to pedestrian environments and walking in Seoul, Korea	NO	Duplicate records
624	The S+5Ds: Spatial access to pedestrian environments and walking in Seoul, Korea	NO	Duplicate records
625	Interseriality and Different Sorts of Walking: Suggestions for a Relational Approach to Urban Walking	NO	Abstract and keywords
626	Meeting places of the Univer-city: On serendipitous encounters in a growing university area	NO	Abstract and keywords
627	A walk accessibility-based approach to assess crowd management in mass religious gatherings	NO	Abstract and keywords
628	ENERGIZING STREET LIFE OF URBAN NEIGHBORHOODS QUALITATIVE NARRATIVES VERSUS QUANTITATIVE METRICS	NO	Abstract and keywords
629	The building blocks of walkability: Pedestrian activity in Abu Dhabi city center	NO	Abstract and keywords
630	Large-scale agent-based simulation model of pedestrian traffic flows	NO	Abstract and keywords
631	Does urban design drive sympathy for the far right?	NO	Abstract and keywords
632	The evaluation of the integration of industrial heritage areas to urban landscape: The case study of sumerbank kayseri cotton factory	NO	Abstract and keywords
633	Tradition, Transformation, and Re-creation in Two Marketplaces: Souq Al Wakrah and Souq Waqif, Qatar	NO	Abstract and keywords
634	Beyond streets: The role of alleys in Abu Dhabi's and Dubai's network systems	NO	Abstract and keywords
635	Exposure to greenery during children’s home–school walks: Socio-economic inequalities in alternative routes	NO	Abstract and keywords
636	Pedestrian circulation simulation based on Ant Colony System in site analysis	NO	Abstract and keywords
637	Novel trends in SNS customers in food and beverage patronage: An empirical study of metropolitan cities in South Korea	NO	Abstract and keywords
638	The spatial mismatch between foreign direct investment and street networks: Evidence from Hanoi, Vietnam	NO	Abstract and keywords
639	RnR-SMART: Resilient smart city evacuation plan based on road network reconfiguration in outbreak response	NO	Abstract and keywords
640	Safety map: Disaster management road network for urban resilience	NO	Abstract and keywords
641	A tool to predict perceived urban stress in open public spaces	NO	Abstract and keywords
642	Locating death anxieties: End-of-life care and the built environment	NO	Abstract and keywords
643	Urban blocks modelling method	NO	Abstract and keywords
644	Public open space, physical activity, urban design and public health: Concepts, methods and research agenda	NO	Abstract and keywords
645	Traditional and novel walkable built environment metrics and social capital	NO	Abstract and keywords
646	Dog-walking in dense compact areas: The role of neighbourhood built environment	NO	Abstract and keywords
647	City love and neighbourhood resilience in the urban fabric: A microcosmic urbanometric analysis of Rotterdam	NO	Abstract and keywords
648	Mapping relations as a design strategy, physical attraction forces correlation for design thinking	NO	Abstract and keywords
649	Forking path: De-scripting interchange architecture at the Ayalon Crosstown Expressway	NO	Abstract and keywords
650	Road safety studies at micro, meso, and macroscopic levels: A systematic review	NO	Abstract and keywords
651	An opinion regarding the grid layout as a goal and parameter	NO	Abstract and keywords
652	Nurturing healthy and resilient communities: An in-depth bibliometric analysis of walkability within the built environment	NO	Abstract and keywords
653	Effects of built environment on walking at the neighbourhood scale. A new role for street networks by modelling their configurational accessibility?	NO	Abstract and keywords
654	3D-GIS as a platform for visual analysis: Investigating a Pompeian house	NO	Abstract and keywords
655	Planning for a fair and resilient city. An Inclusive Accessibility by Proximity index	NO	Abstract and keywords
656	Valuing access to urban greenspace using non-linear distance decay in hedonic property pricing	NO	Abstract and keywords
657	Defining Street-based Local Area and measuring its effect on house price using a hedonic price approach: The case study of Metropolitan London	NO	Abstract and keywords
658	Measuring urban nature for pedestrian health: Systematic review and expert survey	NO	Abstract and keywords
659	Everyday work: A survey of the 2014 Applied Geography Conference	NO	Abstract and keywords
660	Urban form and seasonal PM2.5 dynamics: Enhancing air quality prediction using interpretable machine learning and IoT sensor data	NO	Abstract and keywords

661	Relationships between physical environments and creativity: A scoping review	NO	Abstract and keywords
662	Mathematical beauty and Palladian architecture: Measuring and comparing visual complexity and diversity	NO	Abstract and keywords
663	Fire risk level prediction of timber heritage buildings based on entropy and XGBoost	NO	Abstract and keywords
664	Illuminating the lifecourse of place in the longitudinal study of neighbourhoods and health	NO	Abstract and keywords
665	The built environment and place attachment: Insights from Japanese cities	NO	Abstract and keywords
666	Elderly residents' uses of fragmented outdoor spaces in public housing estates in Hong Kong——Decoding causality and heat-risk exposure	NO	Abstract and keywords
667	Two dimensional accessibility analysis of metro stations in Xi'an, China	NO	Abstract and keywords
668	Revitalizing historic districts: Identifying built environment predictors for street vibrancy based on urban sensor data	NO	Abstract and keywords
669	The relationship between centrality and land use patterns: Empirical evidence from five Chinese metropolises	NO	Abstract and keywords
670	Unveiling fine-scale urban third places for remote work using mobile phone big data	NO	Abstract and keywords
671	Investigating the impacts of urban built environments on users of multiple services in elderly care facilities	NO	Abstract and keywords
672	Greener is healthier: Unraveling the distinction of health impacts of urban green indicators in Wuhan, China	NO	Abstract and keywords
673	Improving the attractiveness and accessibility of campus green space for developing a sustainable university environment	NO	Abstract and keywords
674	Community built environment and the associated ischemic heart disease risk: Evidence from multi-source data in Wuhan, China	NO	Abstract and keywords
675	Transit oriented development among metro station areas in Shanghai, China: Variations, typology, optimization and implications for land use planning	NO	Abstract and keywords
676	Using an improved 3SFCA method to assess inequities associated with multimodal accessibility to green spaces based on mismatches between supply and demand in the metropolitan of Shanghai, China	NO	Abstract and keywords
678	The more walkable, the more livable? -- can urban attractiveness improve urban vitality?	NO	Abstract and keywords
679	Responding to "city cooling action": Assessing and visualizing community-level urban park cooling service performance in Nanjing, China	NO	Abstract and keywords
680	On measuring walking accessibility: A link-based utility approach	NO	Abstract and keywords
681	Spatial configuration and physical quality of linear urban parks: the case of Parque Parahyba I, João Pessoa – PB	NO	Abstract and keywords
682	Metro systems and urban development: Impacts and implications	NO	Abstract and keywords
683	An edge-weighted graph triumvirate to represent modular building layouts	NO	Abstract and keywords
684	What's in a Building? A Descriptive Survey of Adult Inpatient Rehabilitation Facility Buildings in Victoria, Australia	NO	Abstract and keywords
685	What do we visually focus on in a World Heritage Site? A case study in the Historic Centre of Prague	NO	Abstract and keywords
686	The mechanism of block form diversification in urban morphological transformation: Case study of grid blocks in Beijing, China	NO	Abstract and keywords
687	Research on Automatic Generation of Park Road Network Based on Skeleton Algorithm	NO	Abstract and keywords
688	Measuring patterns and mechanism of greenway use – A case from Guangzhou, China	NO	Duplicate records
689	Measuring patterns and mechanism of greenway use - A case from Guangzhou, China	NO	Duplicate records
690	Construction of a comprehensive evaluation model for old community renewal in Suzhou based on smart city concepts	NO	Abstract and keywords
691	Isolated or integrated? Planning and management of urban renewal for historic areas in Old Beijing city, based on the association network system	NO	Abstract and keywords
692	Understanding fly-tipping in urban areas: A social-economic-spatial combinatorial approach enabled by geographically weighted random forest	NO	Abstract and keywords
693	The role of security and walkability in subjective wellbeing: A multigroup analysis among different age cohorts	NO	Abstract and keywords
694	New Tools and Strategies for Design and Operation of Urban Transport Interchanges	NO	Abstract and keywords
695	Evaluating neighbourhood roads through agent-based modelling: A step towards the optimal pedestrian desire path system	NO	Abstract and keywords
696	An elastic urban morpho-blocks (EUM) modeling method for urban building morphological analysis and feature clustering	NO	Abstract and keywords
697	Development of a parametric optimization concept for the sun control of the outdoor spaces: the case study of 5A business park, Cairo, Egypt	NO	Duplicate records
698	Development of a parametric optimization concept for the sun control of the outdoor spaces: the case study of 5A business park, Cairo, Egypt	NO	Duplicate records
699	Vulnerable road users' crash hotspot identification on multi-lane arterial roads using	NO	Abstract and keywords

	estimated exposure and considering context classification		
700	Municipal decision-making for sustainable transportation: Towards improving current practices for street rejuvenation in Canada	NO	Abstract and keywords
701	Pedestrian safety in Kandy Heritage City, Sri Lanka: Lessons from World Heritage Cities	NO	Abstract and keywords
702	An Integrated Model of Achieving Social Sustainability in Urban Context through Theory of Affordance	NO	Abstract and keywords
703	The influence of spatial configuration on pedestrian movement behaviour in commercial streets of low-density cities	NO	Abstract and keywords
704	Taming the road, tamed by the road: sense of road as place among Indigenous Bedouin in an ethnic frontier in Israel	NO	Abstract and keywords
705	Impacts of parking and accessibility on retail-oriented city centres	NO	Abstract and keywords
706	A Big Data Analytics Method for Tourist Behaviour Analysis	NO	Abstract and keywords
707	Using triangulation to assess a suite of tools to measure community severance	NO	Abstract and keywords
708	The influence of street network configuration on sexual harassment patterns in Cairo	NO	Abstract and keywords
709	Benchmarking real and ideal cities - a multicriteria analysis of city performance based on urban form	NO	Abstract and keywords
710	Evaluation of the policy-driven ecological network in the Three-North Shelterbelt region of China	NO	Abstract and keywords
711	Developing a 15-minute city: A comparative study of four Italian Cities-Cagliari, Perugia, Pisa, and Trieste	NO	Duplicate records
712	Developing a 15-minute city: A comparative study of four Italian Cities-Cagliari, Perugia, Pisa, and Trieste	NO	Duplicate records
713	Analysis of urban configuration influence on spatial distribution of criminality in Caceres, Mato Grosso	NO	Abstract and keywords
714	How to develop the walking environment for its ‘consumers’? A conjoint answer derived from people's perception of link and network	NO	Abstract and keywords
715	Urban cohesion vis-à-vis organic spatialization of “Third places” in Saudi Arabia: The need for an alternative planning praxis	NO	Abstract and keywords
716	Associations between neighbourhood built characteristics and sedentary behaviours among Canadian men and women: findings from Alberta's Tomorrow Project	NO	Abstract and keywords
717	Conceptualising the built environment to inform sustainable urban transitions	NO	Abstract and keywords
718	Land use and public transport integration in small cities and towns: Assessment methodology and application	NO	Abstract and keywords
719	How do crosswalk delays affect pedestrian access in zoning areas? Walking access reduction by signalized crosswalks in Seoul, South Korea	NO	Abstract and keywords
720	REDEFINING PLACE FOR WALKING<br>A LITERATURE REVIEW AND KEY-ELEMENTS CONCEPTION	NO	Abstract and keywords
721	Setting the census household into its urban context<br>Visualizations from 19 century Montreal	NO	Abstract and keywords
722	Decoding the Spatial Integration and Morphological Components in Unplanned Squares: the Case of Mugla Saburhane Square	NO	Abstract and keywords
723	How ecosystems services drive urban growth: Integrating nature-based solutions	NO	Abstract and keywords
724	The interactive process of cultural landscape: Taking the trails around Zhengqi Pavilion as an example	NO	Abstract and keywords
725	Urban mobility evolution and the 15-minute city model: from holistic to bottom-up approach	NO	Abstract and keywords
726	Toward human-centric urban infrastructure: Text mining for social media data to identify the public perception of COVID-19 policy in transportation hubs	NO	Abstract and keywords
727	Quality assessment of residential layout designs generated by relational Generative Adversarial Networks (GANs)	NO	Abstract and keywords
728	An investigation into the former consulate of Britain as one of the first samples of modern architecture in Iran	NO	Abstract and keywords
729	Comparing accessibility and connectivity metrics derived from dedicated pedestrian networks and street networks in the context of Asian cities	NO	Abstract and keywords
730	Big geo-data unveils influencing factors on customer flow dynamics within urban commercial districts	NO	Abstract and keywords
731	Evaluating sustainability and land use integration of BRT stations via extended node place model, an application on BRT stations of Tehran	NO	Abstract and keywords
732	SPATIAL CONFIGURATION OF TRADITIONAL HOUSES AND APARTMENT UNIT PLANS IN HO CHI MINH CITY, VIETNAM: A COMPARATIVE STUDY	NO	Abstract and keywords
733	Frozen motion: Contextualizing wheel rut data within and beyond the Pompeiian street grid	NO	Abstract and keywords
734	Resilience and sustainability assessment of cultural heritage and built environment: The Libertad pedestrian walkway in Valdivia, Chile	NO	Abstract and keywords

735	Investigating urban form, and walkability measures in the new developments. The case study of Garnizon in Gdansk	NO	Abstract and keywords
736	Promoting the health of workers through office layout: A scoping literature review	NO	Abstract and keywords
737	To go where no man has gone before: Virtual reality in architecture, landscape architecture and environmental planning	NO	Abstract and keywords
738	Practical simulation of virtual crowds using points of interest	NO	Abstract and keywords
739	Impact of circulation complexity on hospital wayfinding behavior (Case study: Milad 1000-bed hospital, Tehran, Iran)	NO	Abstract and keywords
740	Planning an adaptive reuse development of underutilized urban underground infrastructures: A case study of Qingdao, China	NO	Abstract and keywords
741	A review of research methods on highest and best use for toll rest area	NO	Abstract and keywords
742	Is it really too far? Overestimating walk time and distance reduces walking	NO	Abstract and keywords
743	Determinants of sustainable mode choice in different socio-cultural contexts: A comparison of Rome and San Francisco	NO	Abstract and keywords
744	Enhancing station level Direct-Demand models with Multi-Scalar accessibility indicators	NO	Abstract and keywords
745	Re-examining the role of street network configuration on bicycle commuting using crowdsourced data	NO	Abstract and keywords
746	Street-level built environment on SARS-CoV-2 transmission: A study of Hong Kong	NO	Abstract and keywords
747	Monitoring flood risk evolution: A systematic review	NO	Abstract and keywords
748	Interventions in urban geopolitics	NO	Abstract and keywords
749	Measuring streetscape perceptions from driveways and sidewalks to inform pedestrian-oriented street renewal in Düsseldorf	NO	Abstract and keywords
750	Mapping the impact of ICU design on patients, families and the ICU team: A scoping review	NO	Abstract and keywords
751	Evidence-based neighborhood greening and concomitant improvement of urban heat environment in the context of a world heritage site - Malacca, Malaysia	NO	Abstract and keywords
752	Pocket settings for enhancing social learning experience on campus ground: A verbal-visual preference survey	NO	Abstract and keywords
753	Urban resilience at eye level: Spatial analysis of empirically defined experiential landscapes	NO	Abstract and keywords
754	Recreating historical contexts: methods and strategies for the restoration of the 'spatio-temporal landscape' of Ao Garden in Xiamen, Fujian Province, China	NO	Abstract and keywords
755	SPATIAL CONSTRUCTION, FORM AND EFFECTIVENESS ANALYSIS OF LARGE-SCALE WATERFRONT PARK SYSTEM IN ISLAND-TYPE CITIES (THE CASE OF XIAMEN CITY OF CHINA)	NO	Abstract and keywords
756	Colonial moorings on spatial planning of Mozambique	NO	Abstract and keywords
757	Bibliometric analysis and review of Building Information Modelling literature published between 2005 and 2015	NO	Abstract and keywords
758	A multiscale classification of urban morphology	NO	Abstract and keywords
759	A scoping review on cycling network connectivity and its effects on cycling	NO	Abstract and keywords
760	Human centric accessibility graph for environment analysis	NO	Abstract and keywords
761	Tracking cruise passengers' consumption: An analysis of the relationships between onshore mobility and expenditure	NO	Abstract and keywords
762	Emotions elicited by neighbour noise and coping process: A social media research based on two newspaper articles	NO	Abstract and keywords
763	Assessment of open spaces in inland medium-sized cities of eastern Andalusia (Spain) through complementary approaches: spatial-configurational analysis and decision support	NO	Abstract and keywords
764	Angular and Metric Distance in Road Network Analysis: A nationwide correlation study	NO	Abstract and keywords
765	Associations of children's mental wellbeing and the urban form characteristics of their everyday places	NO	Abstract and keywords
766	The role of turns in pedestrian route choice: A clarification	NO	Abstract and keywords
767	Pedestrian-oriented development in Beirut: A framework for estimating urban design impacts on pedestrian flows through modeling, participatory design, and scenario analysis	NO	Abstract and keywords
768	Measuring security in the built environment: Evaluating urban vulnerability in a human-scale urban form	NO	Abstract and keywords
769	Users as co-designers: Visual–spatial experiences at Whitworth Art Gallery	NO	Abstract and keywords
770	Resilient urban forms: A review of literature on streets and street networks	NO	Abstract and keywords
771	Urban greenways: Operationalizing design syntax and integrating mathematics and science in design	NO	Abstract and keywords
772	Modelling children’s independent territorial range by discretionary and	NO	Abstract and keywords

	nondiscretionary trips		
773	Association between the built environment and children's independent mobility: A meta-analytic review	NO	Abstract and keywords
774	The impact of topological properties of built environment on children independent mobility: A comparative study between discretionary vs. nondiscretionary trips in Dhaka	NO	Abstract and keywords
775	The proportion of independent to dependent trips: A new measure to understand the effects of the built environment on children independent mobility	NO	Abstract and keywords
776	Development and validity of a virtual street walkability audit tool for pedestrian route choice analysis—SWATCH	NO	Abstract and keywords
777	Objective vs. subjective measures of street environments in pedestrian route choice behaviour: Discrepancy and correlates of non-concordance	NO	Abstract and keywords
778	Urban function connectivity: Characterisation of functional urban streets with social media check-in data	NO	Abstract and keywords
779	The economic value of streets: mix-scale spatio-functional interaction and housing price patterns	NO	Abstract and keywords
780	A review of simulation-based urban form generation and optimization for energy-driven urban design	NO	Abstract and keywords
781	A Green View Index Improvement Program for Urban Roads Using a Green Infrastructure Theory - Focused on Chengdu City, Sichuan Province, China	NO	Abstract and keywords
782	Automated generation of versatile data model for analyzing urban architectural void	NO	Abstract and keywords
783	Factors Affecting Walkability of Neighborhoods	NO	Abstract and keywords
784	Estimating generalized measures of local neighbourhood context from multispectral satellite images using a convolutional neural network	NO	Abstract and keywords
785	Varying influences of the built environment on daily and hourly pedestrian crossing volumes at signalized intersections estimated from traffic signal controller event data	NO	Abstract and keywords
786	Residential crimes and neighbourhood built environment: Assessing the effectiveness of crime prevention through environmental design (CPTED)	NO	Abstract and keywords
787	The impact of neighborhood permeability on residential burglary risk: A case study in Seattle, USA	NO	Abstract and keywords
788	Analysis of spatially varying relationships between urban environment factors and land surface temperature in Mashhad city, Iran	NO	Abstract and keywords
789	Effects of planning variables on urban traffic noise at different scales	NO	Abstract and keywords
790	Unraveling the effects of micro-level street environment on dockless bikeshare in Ithaca	NO	Abstract and keywords
791	Investigating pedestrian behaviour in urban environments: A Wi-Fi tracking and machine learning approach	NO	Abstract and keywords
792	Where to improve pedestrian streetscapes<br>Prioritizing and mapping street-level walkability interventions in Cape Town's city centre	NO	Abstract and keywords
793	Mapping the character of urban districts: The morphology, land use and visual character of Chinatowns	NO	Abstract and keywords
794	Critical impact factors of pedestrians traffic combining multiple data sources in Athens	NO	Abstract and keywords
795	Deciphering the influence of TOD on metro ridership: An integrated approach of extended node-place model and interpretable machine learning with planning implications	NO	Abstract and keywords
796	Auditing street walkability and associated social inequalities for planning implications	NO	Abstract and keywords
797	Contemplating museums' service failure: Extracting the service quality dimensions of museums from negative on-line reviews	NO	Abstract and keywords
798		NO	Abstract and keywords
799	Associations of local-area walkability with disparities in residents' walking and car use	NO	Abstract and keywords
800	Evaluation and diagnosis for the pedestrian quality of service in urban riverfront streets	NO	Abstract and keywords
801	Research on influencing factors of travel in underground space based on multi-source data: Spatial optimization design for low-carbon travel	NO	Abstract and keywords
802	Understanding associations between neighbourhood-environment perceptions and walking behaviour in low-tier Chinese cities	NO	Abstract and keywords
803	Three-pronged sustainability assessment of ten towns in the vicinity of Bangkok, Thailand	NO	Abstract and keywords
804	A data-informed analytical approach to human-scale greenway planning: Integrating multi-sourced urban data with machine learning algorithms	NO	Abstract and keywords
805	Unraveling the accessibility-usage mismatch: Identifying driving factors and weather-sensitive metro stations using GPS data for improved metro competitiveness	NO	Abstract and keywords
806	How consumer preference determines site selection in a metropolitan setting: Analysis of retailer perspective to stay ahead of the competition in the aftermath of a large-scale	NO	Abstract and keywords

	crisis		
807	What matters when it comes to “Walk and the city”? Defining a weighted GIS-based walkability index	NO	Abstract and keywords
808	Exploring the relationship between multilevel highway networks and local development patterns—a case study of Taiwan	NO	Abstract and keywords
809	Development of view potential metrics and the financial impact of views on office rents	NO	Abstract and keywords
810	The extended node-place model at the local scale: Evaluating the integration of land use and transport for Lisbon's subway network	NO	Abstract and keywords
811	Pedestrian and transit accessibility on a micro level<br>Results and challenges	NO	Abstract and keywords
812	Hortus Ager Pauperis Erat : Subsistence and Commercial Production in the Urban Gardens of First Century Ad Pompeii	NO	Abstract and keywords
813	Do Jane Jacobs's conditions fostering the presence of people influence crimes in public space? An econometric analysis in la Mariscal neighborhood in Quito	NO	Abstract and keywords
814	Shape grammars overview and assessment for transport and urban design<br>Review, terminology, assessment, and application	NO	Duplicate records
815	Shape grammars overview and assessment for transport and urban design<br>Review, terminology, assessment, and application	NO	Duplicate records
816	How safe do you feel? – A large-scale survey concerning the subjective safety associated with different kinds of cycling lanes	NO	Abstract and keywords
817	Crash risk and subjective risk perception during urban cycling: Evidence for congruent and incongruent sources	NO	Abstract and keywords
818	School route safety perceptions of primary school children and their parents: Effects of transportation mode and infrastructure	NO	Abstract and keywords
819	Trace analysis using Wi-Fi probe positioning and virtual reality for commercial building complex design	NO	Abstract and keywords
820	A study on native and constructed localities in the modern adaptation of villages	NO	Abstract and keywords
821	Flow-based accessibility measurement: The Place Rank approach	NO	Abstract and keywords
822	The Development of comprehensive evaluation indicators of street greenery and the identification of priority greening Areas: Providing more accurate greening planning	NO	Abstract and keywords
823	Designing bike-friendly cities: Interactive effects of built environment factors on bike-sharing	NO	Abstract and keywords
824	Study on slow traffic evaluation method of large hospitals in old urban areas based on Extentics	NO	Abstract and keywords
825	Parametrising historical Chinese courtyard-dwellings: An algorithmic design framework for the digital representation of Siheyuan iterations based on traditional design principles	NO	Abstract and keywords
826	Research on Environmental Behavior of Urban Parks in the North of China during Cold Weather—Nankai Park as a Case Study	NO	Duplicate records
827	Research on Environmental Behavior of Urban Parks in the North of China during Cold Weather—Nankai Park as a Case Study	NO	Duplicate records
828	The prevention strategies for strengthening the resilience of urban high-rise and high-density built environment based on multi-objective optimization: An empirical study in Guangzhou, China	NO	Abstract and keywords
829	The Pueblo Bonito mounds: Formation history, architectural context and representational fields	NO	Abstract and keywords
830	Economic benefits of urban streetscapes: Analyzing the interrelationships between visual street environments and single-family property values in Seoul, Korea	NO	Abstract and keywords
831	What’s your type? A taxonomy of pedestrian route choice behaviour in cities	NO	Abstract and keywords
832	Using street view images to examine the association between human perceptions of locale and urban vitality in Shenzhen, China	NO	Abstract and keywords
833	Ripples create waves: Assessing the catalytic effects of neighborhood micro-regeneration by integrating interpretable machine learning and econometrics	NO	Abstract and keywords
834	The effects of TOD on economic vitality in the post-COVID-19 era	NO	Abstract and keywords
835	Nonlinear and synergistic effects of TOD on urban vibrancy: Applying local explanations for gradient boosting decision tree	NO	Abstract and keywords
836	Bayesian approach to model pedestrian crashes at signalized intersections with measurement errors in exposure	NO	Abstract and keywords
834	Evaluation of the university campus layout for the dual use as a city-level emergency shelter	NO	Abstract and keywords
835	Emergency evacuation shelter management and online drill method driven by real scene 3D model	NO	Abstract and keywords
836	The complexity of property rights embedded in the rural-to-urban resettlement of China: A case of Hangzhou	NO	Abstract and keywords
837	Yang, Juncheng	NO	Abstract and keywords

	Rong, Helena<br>Kang, Yuhao<br>Zhang, Fan<br>Chegut, Andrea		
838	Between morphology and function: How syntactic centers of the Beijing city are defined	NO	Abstract and keywords
839	Research on urban landscape big data information processing system based on ordinary differential equations	NO	Abstract and keywords
840	Does area type matter for pedestrian distribution? Testing movement economy theory on gated and non-gated housing estates in Wuhan, China	NO	Abstract and keywords
841	Enhancing non-motorist safety by simulating trip exposure using a transportation planning approach	NO	Abstract and keywords
842	Measuring daily accessed street greenery: A human-scale approach for informing better urban planning practices	NO	Abstract and keywords
843	Polygonization method for automatic generation of indoor and outdoor pedestrian navigation path for smart city	NO	Abstract and keywords
844	Predicting and Visualizing Human Soundscape Perception in Large-Scale Urban Green Spaces: A Case Study of the Chengdu Outer Ring Ecological Zone	NO	Abstract and keywords
845	Understanding pedestrian behavior and spatial relations: A pedestrianized area in Besiktas, Istanbul	NO	Abstract and keywords
846	Exploring multidimensional aspects of walkability: An innovative analysis approach in Besiktas, Istanbul	NO	Abstract and keywords
847	Relationship between street-connectivity and multi-family residential parcel values Comparison of two urban development projects in Korea	NO	Abstract and keywords
848	Analysis of influence of seismic impact on evacuability in subway stations	NO	Abstract and keywords
849	Impact analysis of street space quality on pedestrian behavior using mobile probe data	NO	Abstract and keywords
850	Examining the correlation between residential environment evaluation and redesign measures to improve disaster prevention performance in densely built-up areas: A case study in the Shinyo neighborhood, Nagata Ward, Kobe	NO	Abstract and keywords
851	CBEES: A fine-grained commercial built environment perception and evaluation based on social media	NO	Abstract and keywords
852	Mindful walks: Understanding the connection between street characteristics and recreational walking routes in older adults with cognitive concerns	NO	Abstract and keywords
853	The negative impacts of human activities on the ecological corridor in the karst highly urbanized area are gradually diminishing: A case study from the karst mountain cities in Southwest China	NO	Abstract and keywords
854	Modelling the effects of street permeability on burglary in Wuhan, China	NO	Abstract and keywords
855	The built environment and mental health among older adults in Dalian: The mediating role of perceived environmental attributes	NO	Abstract and keywords
856	Bayesian spatio-temporal models for mapping urban pedestrian traffic	NO	Abstract and keywords
857	A simple agent-based model for planning for bicycling: Simulation of bicyclists' movements in urban environments	NO	Abstract and keywords
858	Environmental and social sustainability automated evaluation of plazas based on 3D visibility measurements	NO	Abstract and keywords
859	The relationship between street environment and street crime: A case study of Pudong New Area, Shanghai, China	NO	Abstract and keywords
860	Portraying urban landscape: A quantitative analysis system applied in fifteen metropolises in China	NO	Abstract and keywords
861	Simulation study of non-traveling pedestrian traffic in high-speed railway station areas: A case study of the Yangtze River Delta	NO	Abstract and keywords
862	Uncovering how online attractiveness mediates and moderates the impact of multi-scale transportation accessibility on non-work mobility	NO	Abstract and keywords
863	Representing place locales using scene elements	NO	Abstract and keywords
864	Structural renovation of blocks in build-up area of Jiangnan cities, taking Suzhou new district as an example	NO	Abstract and keywords
865	Healthy urban blue space design: Exploring the associations of blue space quality with recreational running and cycling using crowdsourced data	NO	Abstract and keywords
866	Towards a Fairer Green city: measuring unfairness in daily accessible greenery in Chengdu's central city	NO	Abstract and keywords
867	Multi-functional coupling-based rural ecological pattern construction and network prioritization evaluation: A case study of Jiangning District, Nanjing	NO	Abstract and keywords
868	Planning vs Market-led? Identifying urban-suburbs transition zones in metropolitan expansion: A multi-source data fusion framework	NO	Abstract and keywords
869	Analyzing the typology and livability of 15-minute travel at metro stations in high-density cities: A case study of Singapore	NO	Abstract and keywords

870	Public participation in NIMBY risk mitigation: A discourse zoning approach in the Chinese context	NO	Abstract and keywords
871	A social-ecological framework to explore factors associated with transportation walking for rural American adults using case of Mississippi	NO	Abstract and keywords
872	Identifying node-corridor-network of tourist flow and influencing factors using GPS big data: A case study in Gansu and Qinghai provinces, China	NO	Abstract and keywords
873	Exploring walking behaviour and perceived walkability of older adults in London	NO	Abstract and keywords
874	An introduction to connectivity concept and an example of physical connectivity evaluation for underground space	NO	Abstract and keywords
875	A Study on the Hierarchy Analysis for Improving the Utilization of Parks in the Living Area: Case-based on Geumcheon-gu, Seoul, Korea	NO	Abstract and keywords
876	Exploring both home-based and work-based jobs-housing balance by distance decay effect	NO	Abstract and keywords
877	A multiscale walkability assessment approach creating walkable streets: A case study of high-density city, Macau	NO	Abstract and keywords
878	HiVG: A hierarchical indoor visibility-based graph for navigation guidance in multi-storey buildings	NO	Abstract and keywords
879	Street as a big geo-data assembly and analysis unit in urban studies: A case study using Beijing taxi data	NO	Abstract and keywords
880	Nexus of mixed-use vitality, carbon emissions and sustainability of mixed-use rural communities: The case of Zhejiang	NO	Abstract and keywords
881	Understanding of the relation between ethnic diversity and public space<br>A bibliometric analysis	NO	Abstract and keywords
882	Proučevanje povezave med etnično raznolikostjo in javnim prostorom<br>bibliometrična analiza	NO	Abstract and keywords
883	A Study on the Hierarchy Analysis and Utilization Status of Small Parks in the City<br>도심내 소공원의 위계 분석 및 이용현황 분석 연구	NO	Abstract and keywords
884	A Study on Styles of Modern Poetry as Seen through Translation: Focused on Kim Uk’s "The Dance of Mental Anguish(懊惱의 舞蹈)" and Lee Ha-Yoon’s "Scentless Flower Garden(失香의 花園)"	NO	Abstract and keywords
885	The aesthetics of form on park jae-doo's sijo works<br>박재두 시조에 나타난 형식의 미학	NO	Abstract and keywords
886	Evaluation of Park Service in Neighborhood Parks based on the Analysis of Walking Accessibility - Focused on Bundang-gu, Seongnam-si<br>보행접근성 분석에 기반한 근린공원의 공원서비스 평가- 성남시 분당구를 대상으로	NO	Abstract and keywords
887	A Study Crime Vulnerable Elements on University Campuses through the 112 Crime Data Analysis<br>112 범죄 신고 데이터 분석을 통한 캠퍼스 내에서의 범죄취약요소 연구	NO	Abstract and keywords
888	An Analysis on the Characteristics of Factors for the Healing Environment in Healthcare Facilities<br>의료시설의 치유환경조성을 위한 요소별 특성 분석	NO	Abstract and keywords
889	Research for the glocalization of Kid's Cafe Brands based on Kid's Play Contents - focusing on the Kids Cafe in Korea<br>글로컬 브랜드화를 위한 키즈 놀이콘텐츠 연구 - 국내 키즈카페를 중심으로	NO	Abstract and keywords
890	Measurement of Visual Privacy in the Royal Palaces focusing on the Prospect and Refuge<br>전망과 은신처의 개념을 도입한 궁궐건축의 시각적 프라이버시 정량화에 관한 연구	NO	Abstract and keywords
891	A Study on the Space Analysis of SANAA’s Houses based on Space Syntax Theory<br>공간구문론을 이용한 SANAA 주택 작품의 공간 분석 연구	NO	Abstract and keywords
892	A Study on Spatial Use Patterns and Regional Changes of Pedestrian - Centred Public Spaces Near Rail Beds<br>철도부지를 활용한 보행중심 공공공간의 이용행태와 지역의 변화 연구 - 강릉 월화거리를 중심으로 하는 도심지역을 대상으로	NO	Abstract and keywords
893	Exploration on the Sustainability of a Small Community Based on Healthy Community-based Areal Specialization Strategies - Exploring How to Boost Wildflower Village in Gohan-eup, Jeongseon-gun<br>소규모 지역사회의 건강한 공동체 기반 지역특화 전략을 통한 지속가능성의 탐색 - 정선군 고한읍 야생화마을의 활성화 방안 모색을 통하여	NO	Abstract and keywords
894	The Influence of Topological Characteristics and Tourism Environment Information on Spatial Cognition and Satisfaction: Focused on Virtual Reality Techniques<br>공간위상학적 특성과 관광환경정보가 공간 인지와 만족에 미치는 영향:	NO	Abstract and keywords

	가상현실기법을 중심으로		
895	A Study on the Auditory Images of Park Yong-rae's Poetry - Focusing on the Metaphor and Metonymy<br>박용래 시의 청각 이미지 연구 - 은유와 환유를 중심으로	NO	Abstract and keywords
896	The Spatial Semiotics of the East Asian Gardens<br>동아시아 정원의 공간 기호학 연구	NO	Abstract and keywords
897	Relationship between spatially morphology structure and species diversity of plant communities in Karst mountainous cities: A case study of Anshun city	NO	Abstract and keywords
898	The spatial configuration characteristics of classical private gardens in China<br>중국 고전 사가원림(私家园林)의 공간구성 특성에 관한 연구	NO	Abstract and keywords
899	The Characteristic of The Bike Service Area in Han River Civil Park<br>한강시민공원 자전거 이용자권역 특성 연구	NO	Abstract and keywords
900	Spatial characteristics of modern private gardens in Nanxunbased on the optimized space syntax	NO	Abstract and keywords
901	Construction And Poetic Effect of Park YoungLae' Poem<br>박용래 시의 구조와 시적효과	NO	Not about gardens
902	The impact of space syntax spatial attributes on urban land use in Muscat: Implications for urban sustainability	NO	Not about gardens
903	Muscat City Expansion and Accessibility to the Historical Core: Space Syntax Analysis	NO	Not about gardens
904	3D Space Syntax Analysis: Attributes to Be Applied in Landscape Architecture Projects	NO	Not about gardens
905	Urban park in maia – porto: A case study of application of 'space syntax' to landscape architecture	NO	Not about gardens
906	Space Syntax and buried cities: The case of the Roman town of Falerii Novi (Italy)	NO	Not about gardens
907	Analysis of Narrative Space in the Chinese Classical Garden Based on Narratology and Space Syntax-Taking the Humble Administrator's Garden as an Example	NO	Not about gardens
908	Refugee Children's Access to Play in Meso-Environments: A Novel Approach Using Space Syntax and GIS	NO	Not about gardens
909	Spatial Measurement of Mobility Barriers: Improving the Environment of Community-Dwelling Older Adults in Taiwan	NO	Not about gardens
910	Research on the Spatial Structure of Landscape Architecture from Design Intention to Function Use	NO	Not about gardens
911	An Analysis on Seoullo 7017 in Terms of Spatial Configuration and Pedestrian Movement in Comparison with the High-line Project	NO	Not about gardens
912	Using multiple hybrid spatial design network analysis to predict longitudinal effect of a major city centre redevelopment on pedestrian flows	NO	Not about gardens
913	Examining control, centrality and flexibility in Palladio's villa plans using space syntax measurements	NO	Not about gardens
914	Unfolding the dynamical structure of Lisbon's public space: space syntax and micromobility data	NO	Not about gardens
915	The configurational approach to measure the impact of green spaces on urban landscape	NO	Not about gardens
916	Urban landscape accessibility evaluation model based on GIS and spatial analysis	NO	Not about gardens
917	Enhancing outdoor campus design by utilizing space syntax theory for social interaction locations	NO	Not about gardens
918	Structural analysis of the elements of Lynch's image of the city based on space syntax	NO	Not about gardens
919	Spatial social interaction: An explanatory framework of urban space vitality and its preliminary verification	NO	Not about gardens
920	Assessment of the street space quality in the metro station areas at different spatial scales and its impact on the urban vitality	NO	Not about gardens
921	Accessibility and street network characteristics of Urban public facility spaces: Equity research on parks in Fuzhou City based on gis and space syntax model	NO	Not about gardens
922	Reading the urban socio-spatial network through space syntax and geo-tagged Twitter data	NO	Not about gardens
923	Environmental Justice in Greater Los Angeles: Impacts of Spatial and Ethnic Factors on Residents' Socioeconomic and Health Status	NO	Not about gardens
924	The influence of urban spatial structure on building carbon emissions at the neighborhood scale considering spatial effect	NO	Not about gardens
925	A tool to predict perceived urban stress in open public spaces	NO	Not about gardens
926	Natural movement: A space syntax theory linking urban form and function with walking for transport	NO	Not about gardens
927	Latent Dirichlet Allocation (LDA) topic models for Space Syntax studies on spatial experience	NO	Not about gardens
928	The Role of Landscaping Design in Urban Landscape Design in the Context of Big Data	NO	Not about gardens

929	Spatial Characteristics of Suburban Villages Based on Spatial Syntax	NO	Not about gardens
930	Influencing factors of spatial vitality in underground space around railway stations: A case study in Shanghai	NO	Not about gardens
931	Pedestrian vitality characteristics in pedestrianized commercial streets-considering temporal, spatial, and built environment factors	NO	Not about gardens
932	Mapping landscape spaces: Methods for understanding spatial-visual characteristics in landscape design	NO	Not about gardens
933	Spatial-interaction network analysis of built environmental influence on daily public transport demand	NO	Not about gardens
934	Neglected vertical linkage: A study on the form of the canal network in the Huainan Salt Area during the Ming and Qing dynasties using space syntax measurements	NO	Not about gardens
935	Capturing socio-spatial inequality in planetary urbanisation: A multi-dimensional methodological framework	NO	Not about gardens
936	Unveiling the potential of space syntax approach for revitalizing historic urban areas: A case study of Yushan Historic District, China	NO	Not about gardens
937	Evaluation of spatial performance of metro-led urban underground public space: A case study in Shanghai	NO	Not about gardens
938	Narratives in Mamluk architecture: Spatial and perceptual analyses of the madrassas and their mausoleums	NO	Not about gardens
939	Evidence for urban design and public health policy and practice: Space syntax metrics and neighborhood walking	NO	Not about gardens
940	Spatial navigation and place imageability in sense of place	NO	Not about gardens
941	The heterarchical life and spatial analyses of the historical Buddhist temples in the Chiang Saen Basin, Northern Thailand	NO	Not about gardens
942	Role of local climate zone and space syntax on land surface temperature (case study: Tehran)	NO	Not about gardens
943	Understanding the role of urban form in explaining transportation and recreational walking among children in a logistic GWR model: A spatial analysis in Istanbul, Turkey	NO	Not about gardens
944	Behavioral responses of the elderly regarding spatial configuration: An elderly care institution case study	NO	Not about gardens
945	Spatial distributive effects of public green space and COVID-19 infection in London	NO	Not about gardens
946	Spatial analysis of various multiplex cinema types	NO	Not about gardens
947	Crime prevention in urban spaces through environmental design: A critical UK perspective	NO	Not about gardens
948	Exploring Urban Service Location Suitability: Mapping Social Behavior Dynamics with Space Syntax Theory	NO	Duplicate records
949	Exploring Urban Service Location Suitability: Mapping Social Behavior Dynamics with Space Syntax Theory	NO	Duplicate records
950	The spatio-temporal evolution and transformation mode of human settlement quality from the perspective of “production-living-ecological" spaces--a case study of Jilin Province	NO	Not about gardens
951	Benches, fountains and trees: Using mixed-methods with questionnaire and smartphone data to design urban green spaces	NO	Not about gardens
952	Assessing the morphological distribution of urban green spaces for the future sustainable greenery planning: a case study of Penang, Malaysia	NO	Duplicate records
953	Assessing the morphological distribution of urban green spaces for the future sustainable greenery planning: a case study of Penang, Malaysia	NO	Duplicate records
954	Investigating the Impact of Environmental Factors on Electricity Consumption Using Spatial Data Mining and Artificial Neural Network: A Case Study in Yazd City	NO	Not about gardens
955	Spatial structure of workplace and communication between colleagues: A study of E-mail exchange and spatial relatedness on the MIT campus	NO	Not about gardens
956	Shortest path distance vs. least directional change: Empirical testing of space syntax and geographic theories concerning pedestrian route choice behaviour	NO	Not about gardens
957	Street network morphology and active mobility to school: Applying space syntax methodology in Shiraz, Iran	NO	Not about gardens
958	Unraveling the relative contribution of TOD structural factors to metro ridership: A novel localized modeling approach with implications on spatial planning	NO	Not about gardens
959	Effects of 3D urban morphology on CO2 emissions using machine learning: Towards spatially tailored low-carbon strategies in Central Wuhan, China	NO	Not about gardens
960	Space–use analysis through computer vision	NO	Not about gardens
961	Main challenges and opportunities to dynamic road space allocation: From static to dynamic urban designs	NO	Not about gardens
962	Measuring residents' perceptions of city streets to inform better street planning through deep learning and space syntax	NO	Not about gardens

963	Effects of open space accessibility and quality on older adults' visit: Planning towards equal right to the city	NO	Not about gardens
964	CVTLayout: Automated generation of mid-scale commercial space layout via Centroidal Voronoi Tessellation	NO	Not about gardens
965	Response of habitat quality to urban spatial morphological structure in multi-mountainous city	NO	Not about gardens
966	Formalising the urban pattern language: A morphological paradigm towards understanding the multi-scalar spatial structure of cities	NO	Not about gardens
967	Understanding the impacts of space design on local outdoor thermal comfort: An approach combining DepthmapX and XGBoost	NO	Not about gardens
968	Understanding the relationship between the spatial configuration and the crime rate of Downtown Eastside in Vancouver, Canada	NO	Not about gardens
969	Evaluation of commercial space accessibility in tourist town: A case research of Maotai Town in China	NO	Not about gardens
970	Quantitative analysis of spatial vitality and spatial characteristics of urban underground space (UUS) in metro area	NO	Not about gardens
971	The spatial vitality and spatial environments of urban underground space (UUS) in metro area based on the spatiotemporal analysis	NO	Not about gardens
972	Uncovering the relationship among spatial vitality, perception, and environment of urban underground space in the metro zone	NO	Not about gardens
973	Urbanization through resettlement and the production of space in Hangzhou's concentrated resettlement communities	NO	Not about gardens
974	Measuring the accessibility deprivation of concentrated resettlement communities in China: An integrated approach of space syntax and multi-criteria decision analysis	NO	Not about gardens
975	Global and local associations between urban greenery and travel propensity of older adults in Hong Kong	NO	Not about gardens
976	Applying and exploring a new modeling approach of functional connectivity regarding ecological network: A case study on the dynamic lines of space syntax	NO	Not about gardens
977	Research on urban landscape big data information processing system based on ordinary differential equations	NO	Not about gardens
978	Fuzzy logic in agent-based modeling of user movement in urban space: Definition and application to a case study of a square	NO	Not about gardens
979	Morphological and functional polycentric structure assessment of megacity: An integrated approach with spatial distribution and interaction	NO	Not about gardens
980	Spatio-visual experience of movement through the Yuyuan Garden: A computational analysis based on isovists and visibility graphs	NO	Not about gardens
981	Redefining active mobility from spatial to social in Singapore	NO	Not about gardens
982	Investigating the socio-spatial relations of the built environment using the Space Syntax analysis – A case study of Tirana City	NO	Not about gardens
983	Socioeconomic performance of in-between open spaces in a post-socialist city of Tirana, Albania	NO	Not about gardens
984	Exploration on the spatial spillover effect of infrastructure network on urbanization: A case study in Wuhan urban agglomeration	NO	Not about gardens
985	Evaluating the impact of mass housings' in-between spaces' spatial configuration on users' social interaction	NO	Not about gardens
986	Spatial-temporal evolution characteristics and critical factors identification of urban resilience under public health emergencies	NO	Not about gardens
987	Suitability Analyses between Exercise Patterns of Morning Exercise and Green Space Characteristics: A Case Study of Zhaolin Park, China	NO	Not about gardens
988	Quantitative Estimation of the Internal Spatio–Temporal Characteristics of Ancient Temple Heritage Space with Space Syntax Models: A Case Study of Daming Temple	NO	Duplicate records
989	Quantitative Estimation of the Internal Spatio–Temporal Characteristics of Ancient Temple Heritage Space with Space Syntax Models: A Case Study of Daming Temple	NO	Duplicate records
990	Research on urban spatial structure based on the dual constraints of geographic environment and POI big data	NO	Not about gardens
991	Urban spatial vulnerability analysis based on urban systems using support vector machine	NO	Not about gardens
992	Transmission risks of airborne respiratory infectious disease and their influencing factors in and around urban outdoor recreational spaces	NO	Not about gardens
993	Discussion on the Optimization Method of Public Service Facility Layout from the Perspective of Spatial Equity: A Study Based on the Central City of Shanghai	NO	Not about space syntax
994	Influence of Urban Park Pathway Features on the Density and Intensity of Walking and Running Activities: A Case Study of Shanghai City	NO	Not about space syntax
995	How to lead the optimization of parks spatial patterns more comprehensively with the philosophy of green equity: A case of Chengdu	NO	Not about space syntax

996	Research on Automatic Generation of Park Road Network Based on Skeleton Algorithm	NO	Not about space syntax
997	A rule-based servicescape design support system from the design patterns of theme parks	NO	Not about space syntax
998	Gender disparities in perceived visibility and crime anxiety in piloti parking spaces of multifamily housing: A virtual reality study	NO	Not about space syntax
999	Recreating historical contexts: methods and strategies for the restoration of the 'spatio-temporal landscape' of Ao Garden in Xiamen, Fujian Province, China	NO	Not about space syntax
1000	SPATIAL CONSTRUCTION, FORM AND EFFECTIVENESS ANALYSIS OF LARGE-SCALE WATERFRONT PARK SYSTEM IN ISLAND-TYPE CITIES (THE CASE OF XIAMEN CITY OF CHINA)	NO	Not about space syntax
1001	Integrating GIS, 3D-Isovist, and an NSGA-II multi-objective optimization algorithm for automation of design process in urban parks and public open spaces	NO	Not about space syntax
1002	Antecedents and consequences of park crowding: Linking park attractiveness, perceived crowding, and revisit intention	NO	Not about space syntax
1003	Evaluating the accessibility of urban parks and waterfronts through online map services: A case study of Shaoxing, China	NO	Not about space syntax
1004	Subject Index	NO	Not full text
1005	Environmental Psychology and Human Well-Being	NO	Not full text
1006	Urban Ecology	NO	Not full text
1007	Handbook of Clinical Neurology	NO	Not full text
1008	Livable Streets 2.0	NO	Not full text
1009	Sustainable Energy Transition for Cities	NO	Not full text
1010	Intelligent Environments (Second Edition)	NO	Not full text
1011	Spatial cultures of public libraries : architecture, collective use and political agendas in medellin's library-parks	NO	Not full text
1012	Spatial dimensions of the influence of urban green-blue spaces on human health: A systematic review	NO	Not full text
1013	Socio-Spatial Experience in Space Syntax Research: A PRISMA-Compliant Review	NO	Review article
1014	Socio-spatial segregation and human mobility: A review of empirical evidence	NO	Review article
1015	Associations of public open space attributes with active and sedentary behaviors in dense urban areas: A systematic review of observational studies	NO	Review article
1016	Integrated design of transport infrastructure and public spaces considering human behavior: A review of state-of-the-art methods and tools	NO	Review article
1017	Spatial measures and methods in sustainable urban morphology: A systematic review	NO	Review article
1018	A review of spatial approaches in road safety	NO	Review article
1019	Atlas Río Mayo: Dynamic Territory and Inter-Scalar Waman-Samanas Landscape in the Peruvian Amazon. Case Study: Bajo Mayo Network Communities: Flores del Río Mayo, San Antonio del Río Mayo, Churuyacu del Río Mayo and Solo del Río Mayo. Lamas, San Martin	NO	Dissertation
1020	Kuzey Makedonya'da Tetova şehrinin mekân yapilanmasindaki gizli ağThe Hidden Network in the Spatial Structure of the City of Tetova in North Macedonia	NO	Dissertation
1021	A Historical Inquiry into the Failure of Downtown Eugene's Pedestrian Mall Strategy to Revitalize the Retail Core, 1971–2002	NO	Dissertation
1022	The Utilization of Space Syntax Theories to Develop a Parametric and Generative Urban Design Program in Grasshopper	NO	Dissertation
1023	Transitive Gestures: Everyday Structures at Play	NO	Dissertation
1024	"Seeing" or "Being Seen": Research on the Sight Line<br>Design in the Lion Grove Based on Visitor<br>Temporal–Spatial Distribution and Space Syntax	Yes	
1025	A Study on Accessibility of Urban Park in Hefei City Intergrating Space Syntax and Baidu Heatmaps	Yes	
1026	A Study on the Correlation between Spatial Structure and Utilization of Urban Parks in Cheonan City - Focusing on Sinbuldang and Ssangyong 2-dong<br>천안 도심공원의 공간구조와 이용도의 상관성 연구 - 신불당 및 쌍용 2 동지역을 중심으로	Yes	
1027	Analysis of Narrative Space in the Chinese Classical Garden Based on Narratology and Space Syntax—Taking the Humble Administrator's Garden as an Example	Yes	
1028	Application of space syntax in neighbourhood park research: an investigation of multiple socio-spatial attributes of park use	Yes	
1029	Can trail spatial attributes predict trail use level in urban forest park? An examination integrating GPS data and space syntax theory	Yes	
1030	Combining GPS and space syntax analysis to improve understanding of visitor temporal–spatial behaviour: a case study of the Lion Grove in China	Yes	

1031	Correlations between Spatial Attributes and Visitor Stay in Chinese Gardens: A Case Study of the Ningbo Tianyige Museum Gardens	Yes	
1032	Creating an endless visual space:<br>An Isovist analysis of a small<br>traditional Chinese garden	Yes	
1033	Engaging in social interaction: relationships between the accessibility of path structure and intensity of passive social<br>interaction in urban parks	Yes	
1034	Integrating space syntax with field observations to understand the spatial logic of park infrastructure	Yes	
1035	Measuring the psychological restoration of visitors in urban parks regarding their spatial configuration; Case study: Eram Park in Hamedan	Yes	
1036	Spatial configuration analysis of a traditional garden in Yangzhou city: a comparative case study of three typical gardens	Yes	
1037	Spatio-Temporal Experience of Tour Routes in the Humble Administrator's Garden Based on Isovist Analysis	Yes	
1038	The mathematics of spatial transparency and mystery: using syntactical data to visualise and analyse the properties of the Yuyuan Garden	Yes	
1039	The Impact of Spatial Configuration on Perceived Accessibility of Urban Parks Based on Space Syntax and Users' Responses	Yes	
1040	Enhancing Urban Park Connectivity and User Experience Through Space Syntax Analysis with Environmental Performance Analysis: A Case Study in New Damietta City, Egypt	No	Unpublished pilot study
