# Supplementary material for: The influence of garden spatial configuration on tourist behavior: A systematic review based on Space Syntax
Source: PLoS One. 2026 Jan 2;21(1):e0339994. doi: 10.1371/journal.pone.0339994 (PMC12758741; doi:10.1371/journal.pone.0339994)
Supplement: Attachment — Submitted filename: rebuttal letter.doc [file pone.0339994.s005.doc]

Rebuttal Letter

Title: *The Influence of Garden Spatial Configuration on Tourist Behavior: A Systematic Review Based on Space Syntax*

Dear Editors,

Thank you for your valuable feedback regarding our previous submission (Manuscript ID: PONE-D-25-30762). We sincerely appreciate your constructive comments, which have helped us significantly improve the quality and rigor of our manuscript. Below, we provide a detailed response to the editorial concerns and describe the corresponding revisions made.

**Comment 1:** The manuscript does not meet the methodological standards for a systematic review, including the lack of clearly formulated research questions and limited methodological transparency.

**Response:**

We fully agree with your assessment and have made substantial improvements to ensure methodological rigor. Specifically, we added two clearly formulated research questions (RQ1 and RQ2) in the Introduction section, which guide the structure and purpose of the review.

“RQ1: What are the main research trends from 2016 to 2025 in studies using Space Syntax to explore the relationship between garden spatial configuration and tourist behavior?

RQ2: How has the Space Syntax-based approach been applied to investigate the influence of garden spatial configuration on tourist behavior?

RQ1 focuses on identifying the characteristics and trends of publications from 2016 to 2025, including research contexts and features, countries of origin, publication year trends, and journal sources. RQ2 delves into various aspects of tourist behavior, such as Space Syntax indicators, types of tourist behavior, mediating mechanisms, and their relationship with garden spatial configuration. By comprehensively integrating and analyzing existing research findings, this review enhances the understanding of research dynamics and key discoveries in this field. Moreover, by revealing the limitations and shortcomings of current studies, it offers theoretical support and directional guidance for future research.”

Furthermore, the Results section (Section 3) is explicitly structured around these two questions:

Section 3.2 (Literature sources and publication trends) answers RQ1 by analyzing publication years, country distribution, and research types.

Sections 3.3 to 3.5 respond to RQ2 by summarizing spatial syntax metrics, behavioral effects, and mediating mechanisms of perception.

Finally, in the Discussion section (Section 4), we systematically revisit RQ1 and RQ2 to synthesize the key findings and limitations, thereby ensuring a clear logical connection between research questions, results, and interpretation.

This comprehensive revision enhances the focus, coherence, and analytical clarity of the systematic review.

Additionally, we reorganized the Methods section (Section 2) according to PRISMA 2020 guidelines, and clarified the literature screening, data extraction, and quality appraisal process. Detailed search strategies, screening procedures, and evaluation tools (e.g., PICOS criteria and CCAT scoring) are presented and supported with supplemental tables (S1–S3).

**Comment 2:** The justification for including only English-language studies was insufficient and introduces potential bias.

**Response:**

Thank you for pointing out this important issue. In the revised manuscript, we have removed all language restrictions during the literature search. The updated inclusion criteria now state that studies in all available languages were considered, provided they met the relevance and quality standards. This change is reflected in Section 2.2.

“2.2 There were no restrictions placed on the language of the studies, and literature in any language was included as long as it met the relevance and quality criteria. ”

**Comment 3:** The study does not provide enough theoretical or methodological innovation to justify publication.

**Response:**

We respectfully acknowledge this concern and have revised the manuscript to emphasize its contributions in two key areas:

1. Theoretical Innovation

We have constructed a structured explanatory pathway—“Structure → Perception → Behavior”—based on existing empirical findings. This framework reveals the mediating role of tourist perception in connecting spatial structure and behavior, offering theoretical depth beyond descriptive analysis (Section 4.3).

“This review further reveals that tourist perception serves as a mediating factor between spatial structure and behavioral patterns, constructing a "structure–perception–behavior" pathway. ”

2. Methodological Refinement

We enhanced our systematic review methodology by integrating spatial configuration metrics, perception analysis, and behavioral outcome categorization. Furthermore, the review covers multi-country case studies (China, Korea, Poland, Pakistan) and employs a comprehensive coding matrix (Table 4 and 6), thereby offering generalizable findings and cross-cultural insights.

We sincerely appreciate the opportunity to revise and resubmit our manuscript. We believe that the extensive revisions made in response to the reviewers’ comments have substantially improved the clarity, methodological rigor, and academic value of the paper. We hope that the revised version will meet the standards of PLOS ONE and will be favorably considered for peer review.

Thank you once again for your time and thoughtful evaluation.

Yours sincerely,

Bin Li

Faculty of Human Ecology

Universiti Putra Malaysia

Email: libin.upm0319@gmail.com
