# Supplementary material for: The influence of garden spatial configuration on tourist behavior: A systematic review based on Space Syntax
Source: PLoS One. 2026 Jan 2;21(1):e0339994. doi: 10.1371/journal.pone.0339994 (PMC12758741; doi:10.1371/journal.pone.0339994)
Supplement: Attachment — Submitted filename: Response to Reviewers.docx [file pone.0339994.s006.docx]

Response to Reviewers' Comments

Title: *The Influence of Garden Spatial Configuration on Tourist Behavior: A Systematic Review Based on Space Syntax*

Manuscript ID: PONE-D-25-30762

We sincerely thank the academic editor and reviewers for their valuable feedback. We have addressed all comments and revised the manuscript accordingly. Our detailed responses are provided below.

**Reviewer #1:**

**1.Comment:**While the conceptual pathway is sound, the review relies heavily on synthesizing prior findings without clearly proposing or testing a new theory. The model would benefit from a visual diagram or conceptual map.

**Response:** Thank you for this helpful suggestion. We have added a visual framework to clarify the conceptual pathway and make the synthesis more transparent. The figure is placed at the end of the *Introduction* and labeled **“Fig 1. Flow diagram for literature review.”** It outlines the key constructs and their relationships and summarizes the review process. We believe this diagram strengthens the logic of the model and improves readability.

In addition, we have introduced a mechanism-oriented synthesis in **Section 4.3** “Structure–Perception–Behavior (SPB) framework,” illustrated in **“Fig 6. Structure–Perception–Behavior (SPB) framework.”** This framework articulates how spatial structure (e.g., integration, connectivity, visual integration, isovist area) influences tourist perception (openness/readability, cultural cognition, safety/restoration), which in turn shapes behavioral outcomes such as path choice, dwell, and revisit intention.

**2.Comment:**Despite following PRISMA guidelines and employing the CCAT tool, the scope of analysis is narrow. Only 10 studies are included over a 9-year span. The justification for this small sample should be more robust (based on my knowledge.)

**Response:** Thank you for pointing this out. In response, we have re-screened the studies under **Section 2.3. Study selection**, and the final number of included studies has been updated to 18, in accordance with the predefined inclusion criteria. We have also revised the corresponding flow chart, now presented as “**Fig 2. PRISMA flow diagram.**”

Furthermore, in **Section 2.2. Criteria and Quality assessment**, we have added the following explanation:

“Although the search covered major databases, the number of studies ultimately included was relatively small; therefore, this study does not rely solely on statistical frequencies but adopts an interpretive synthesis, emphasizing the correspondence between ‘metrics–representations–behavior’ and the elucidation of the ‘structure–perception–behavior’ mechanism.”

We believe these revisions provide a more robust justification for the sample size and clarify the methodological rationale.

**3.Comment:**The synthesis could benefit from clearer distinction between quantitative behavioral data (e.g., GPS paths, density) and subjective perceptual data. Future reviews could adopt mixed-method meta-analysis frameworks.

**Response:** Thank you for this constructive comment. In the revised manuscript, we have provided a clearer distinction between quantitative behavioral data and subjective perceptual data. Specifically, in **Section 3.1 “Research characteristics”**, we added **Table 4. Characteristics of the studies**, which systematically summarizes the included studies according to different spatial representations (VGA, Segment, Isovist, Convex) and links them to different types of behavioral outcomes.

For subjective perceptual data, we added a dedicated subsection **“3.5. Tourist perception as a mediating effect”**, which systematically synthesizes perceptual variables such as aesthetic preference, cultural cognition, and safety/restoration. These perceptual variables are explicitly linked to spatial-structural drivers and behavioral outcomes. To complement this, we introduced **Table 8. The impact of tourist perception on behavioral outcomes**, which presents the relationships in a structured manner.

We believe these additions enhance the clarity of the synthesis and address the reviewer’s request.

**4.Comment:**Some terms like 'isovist' or 'visual integration' should be briefly defined when first used.

**Response:** Thank you for pointing this out. In the revised manuscript, we have added brief definitions of key terms when they first appear in **Section 3.3 “Characteristics of space syntax indicators.”** Specifically:

**3.3.1. Depth and Integration**

Specific content added: “Mohamed et al.[30] emphasize that depth refers to the total number of steps required from a spatial unit to other spaces, which can reveal the marginal characteristics of the spatial structure; depth is effective for assessing less-frequented areas…… Conversely, lower integration values indicate that the space lies in peripheral and relatively enclosed areas, with a higher degree of enclosure and concealment[45].”

**3.3.2. Control value and Choice**

Specific content added: “Mohamed et al.[30] indicate that the control value measures the relative control of a spatial node over its adjacent nodes within a spatial network, reflecting the functional mechanism of the focal spatial unit in the local spatial structure and serving to assess its effects on surrounding spatial units…...Higher choice values indicate that the spatial unit plays a central role in route selection within the overall network[45].”

**3.3.3. Connectivity and Intelligibility**

Specific content added: “Wu et al.[31] state that connectivity refers to the number of links from a given spatial unit to its adjacent units, reflecting local connectedness…….Lower values indicate a more complex structure, increasing the difficulty of spatial understanding. Based on the intelligibility value, spaces can be classified into three categories[48] (Table 6). ”

**3.3.4. Isovist Area, Visual Integration and Extended Metrics**

Specific content added: “Benedikt.[49] emphasizes that the isovist area refers to the area of the spatial region fully visible from a given observation point; the larger the isovist area, the broader the visual extent covered by the space and the greater its openness and visibility…….Huang and Lee.[28] examined the integration of Baidu heatmaps with space syntax and found a statistically significant correlation between integration and visitor clustering intensity.”

**5.Comment:**Ensure figures referenced (e.g., PRISMA diagram, Fig. 3) are embedded correctly in final layout.

**Response:** Thank you for this reminder. We have carefully checked all figures in the revised manuscript, to ensure that they are correctly referenced and embedded in the final layout.

**6.Comment:**Writing is mostly clear but would benefit from further proofreading for redundancy and flow.

**Response:** Thank you for this valuable comment. We have carefully proofread the entire manuscript to improve readability, reduce redundancy, and enhance the overall flow of the text. We believe these revisions have significantly improved the clarity and coherence of the paper.

**Reviewer #2:**

**1.Comment:**The manuscript examines a pertinent and timely subject with potential significance for both scholarly inquiry and practical implementation. The study design seems methodologically robust, and the data provided corroborate the overarching conclusions. The statistical analyses utilise suitable methods and seem to have been conducted with sufficient rigour, enhancing the reliability of the findings. The manuscript is predominantly well-composed, with arguments articulated in a coherent and logical fashion; however, minor grammatical revisions and occasional rephrasing could enhance its flow and readability. Certain terms and definitions require more precise clarification to enhance accessibility for readers from varied backgrounds.

**Response:** Thank you for this helpful comment. We have carefully proofread the manuscript and made minor grammatical revisions and wording adjustments to improve clarity, readability, and flow. Additionally, In the revised manuscript, we have provided clearer definitions of the technical terms used in the study. Specifically, these definitions have been added under **Section 3.3 “Characteristics of space syntax indicators”**, including:

**3.3.1. Depth and Integration**

Specific content added: “Mohamed et al.[30] emphasize that depth refers to the total number of steps required from a spatial unit to other spaces, which can reveal the marginal characteristics of the spatial structure; depth is effective for assessing less-frequented areas…… Conversely, lower integration values indicate that the space lies in peripheral and relatively enclosed areas, with a higher degree of enclosure and concealment[45].”

**3.3.2. Control value and Choice**

Specific content added: “Mohamed et al.[30] indicate that the control value measures the relative control of a spatial node over its adjacent nodes within a spatial network, reflecting the functional mechanism of the focal spatial unit in the local spatial structure and serving to assess its effects on surrounding spatial units…...Higher choice values indicate that the spatial unit plays a central role in route selection within the overall network[45].”

**3.3.3. Connectivity and Intelligibility**

Specific content added: “Wu et al.[31] state that connectivity refers to the number of links from a given spatial unit to its adjacent units, reflecting local connectedness…….Lower values indicate a more complex structure, increasing the difficulty of spatial understanding. Based on the intelligibility value, spaces can be classified into three categories[48] (Table 6). ”

**3.3.4. Isovist Area, Visual Integration and Extended Metrics**

Specific content added: “Benedikt.[49] emphasizes that the isovist area refers to the area of the spatial region fully visible from a given observation point; the larger the isovist area, the broader the visual extent covered by the space and the greater its openness and visibility…….Huang and Lee.[28] examined the integration of Baidu heatmaps with space syntax and found a statistically significant correlation between integration and visitor clustering intensity.”

**2.Comment:**Although the methodology is valid, the authors should offer more explicit justification for specific decisions regarding data handling, analysis, or variable selection to enhance the robustness of their approach. It would be advantageous to succinctly emphasise the originality and prospective impact of this research in the introduction, clearly articulating how it enhances current understanding. The manuscript possesses merit and, with minor enhancements in clarity and language refinement, can significantly contribute to the literature.

**Response:** Thank you for this constructive comment. In the revised manuscript, we have provided more explicit justification for the methodological framework. At the end of the **Introduction**, we added **Fig. 1 “Flow diagram for literature review”**, which visually illustrates the conceptual pathway and clarifies how data handling, analysis, and variable selection were systematically conducted. This addition strengthens the transparency and robustness of the methodology. Furthermore, we emphasized the originality and prospective impact of this research in the **Introduction**, Specific content added: “The necessity of this systematic review stems from the current paucity of comprehensive syntheses based on Space Syntax that analyze the interrelationship between garden spatial structure and tourist behavior. Although prior studies have confirmed the correlation between spatial configuration and tourist behavior, a lack of systematic reviews persists—particularly those integrating the explanatory power and adaptability of different spatial variables. ”

**Reviewer #3:**

**1.Comment:**This is an interesting article that accumulates recent developments in the use of spatial configuration metrics to determine patterns in tourist behaviour in gardens. The authors appear to have done a relatively comprehensive collection, final selection and description of research studies relying on existing models and standard processes. The manuscript is readable and intelligible though slightly repetitive. Where the article falters is in going beyond simple enumeration of the methods used in each study particularly as that relates to spatial configuration. While this is indeed an emerging domain with various published papers, the strategy for the review chosen here seems lacking. The authors have chosen to do a relatively shallow reading of the methodology of each article, focusing only on metrics, whereas research that is typically considered part of the space syntax domain has produced a multitude of combinations of spatial representations, algorithms, metrics and other methodological considerations such as radius (the spatial limit to the reach of the various algorithms). This approach would have worked fine if the sample was larger, but there are only 10 studies selected and all use rather different methods of getting to the result, not always equivalent. Because the sample is small, I would have expected a much deeper explanation of the spatial configuration methodologies. For example, a cursory reading of the studies suggests that they use different representations of space, but these are not mentioned in the manuscript. They do not appear as part of the methodology review or identified per-study. Some studies seem to use Visibility Graph Analysis (VGA) (referenced as 13, 15, 30 and 31), some Segment maps (28, 30, 32, 33) some Isovists (27, 31) and some Convex maps (20, 29). This can be problematic in some cases because metrics such as "Integration" and "Connectivity" produce different values for different representations at the same location (especially VGA and Segment maps) making comparison to human behaviour and perception difficult. Given the small sample I would have also at least expected the authors to further provide examples of the use of these methods in other scales of the built environment.

**Response:** Thank you for this thoughtful comment. We have strengthened the review strategy and deepened the methodological synthesis as follows:

a. **Clarifying the review strategy** — We added a visual overview of the research process at the end of the *Introduction*, presented as **Fig. 1 “Flow diagram for literature review.”** This figure makes the review strategy and steps explicit.
b. **Updating the study sample and screening** — In **Section 2.3 “Study selection,”** we re-screened the literature and updated the final sample to **18 studies**, and we revised the PRISMA chart accordingly as **Fig. 2 “PRISMA flow diagram.”** This explicitly documents the sample size and screening pathway.
c. **Deepening the methodological synthesis** — In **Section 3.1 “Research characteristics,”** we added the following content and a new table to move beyond a simple enumeration of metrics:

Specific content added: “This study provides a systematic synthesis of the 18 included studies (Table 4), summarizing the following dimensions: study location; spatial representations (VGA, Segment, Isovist, Axial); space syntax metrics (e.g., Integration, Connectivity, Choice, Control, Depth, Intelligibility); radius/weight settings; and behavioral outcomes. Due to differences in computational foundations and scale semantics across representations, identically named metrics (Integration, Connectivity) may yield different values at the same location and lead to different interpretations, with the discrepancy between VGA and Segment being particularly pronounced. To ensure comparability, we group by representation during synthesis and interpret the relationships between metrics and behavior/perception under similar radius/weight settings, avoiding direct numerical comparisons across representations.”

We also added **Table 4 “Characteristics of the studies.”** which, for each study, records the spatial representation, metrics, radius/weight settings, study sites, and behavioral outcomes, thereby providing a transparent, per-study methodological profile rather than a metric-only listing.

d. **Term definitions to support cross-representation reading — In Section 3.3 “Characteristics of space syntax indicators” (subse**ctions **3.3.1–3.3.4**), we added concise definitions and explanations for core indicators (Depth/Integration; Control/Choice; Connectivity/Intelligibility; Isovist Area/Visual Integration and extended metrics), which further explains why identically named metrics can differ under different spatial representations

**3.3.1. Depth and Integration**

Specific content added: “Mohamed et al.[30] emphasize that depth refers to the total number of steps required from a spatial unit to other spaces, which can reveal the marginal characteristics of the spatial structure; depth is effective for assessing less-frequented areas…… Conversely, lower integration values indicate that the space lies in peripheral and relatively enclosed areas, with a higher degree of enclosure and concealment[45].”

**3.3.2. Control value and Choice**

Specific content added: “Mohamed et al.[30] indicate that the control value measures the relative control of a spatial node over its adjacent nodes within a spatial network, reflecting the functional mechanism of the focal spatial unit in the local spatial structure and serving to assess its effects on surrounding spatial units…...Higher choice values indicate that the spatial unit plays a central role in route selection within the overall network[45].”

**3.3.3. Connectivity and Intelligibility**

Specific content added: “Wu et al.[31] state that connectivity refers to the number of links from a given spatial unit to its adjacent units, reflecting local connectedness…….Lower values indicate a more complex structure, increasing the difficulty of spatial understanding. Based on the intelligibility value, spaces can be classified into three categories[48] (Table 6). ”

**3.3.4. Isovist Area, Visual Integration and Extended Metrics**

Specific content added: “Benedikt.[49] emphasizes that the isovist area refers to the area of the spatial region fully visible from a given observation point; the larger the isovist area, the broader the visual extent covered by the space and the greater its openness and visibility…….Huang and Lee.[28] examined the integration of Baidu heatmaps with space syntax and found a statistically significant correlation between integration and visitor clustering intensity.”

e. **Stating the limitation and our representation-aware strategy (Section 5 “Limitations”)** To make our review strategy explicit and acknowledge the boundary conditions, we revised **Section 5 Limitations** as follows:

Specific content added: “Due to inconsistencies in computation and scale semantics across different spatial representations, we do not perform direct numerical comparisons across representations; instead, we examine the associations between metrics and behavior/perception within the same representation and under comparable radius/weight settings. Although we enumerated typical application contexts of space syntax at other scales—such as building interiors, campuses/parks, and urban street networks—to strengthen the methodological background, the scope of this review remains confined to the garden scale.”

**2.Comment:** Gardens are at the "small urban area" scale, meaning that the authors could have examined papers from both smaller scales (buildings such as offices, schools and hospitals, usually employing VGA) and larger scales (large urban areas and cities typically employing segment analysis).

**Response:** Thank you for this valuable suggestion. To address the request for cross-scale context, we have added a new subsection in the Discussion: **“4.4. Cross-scale methodological implications for garden spatial research.”**

Specific content added: “This review centers on garden spatial configuration，emphasizing gardens as a typical "small-scale urban space." By contrast, other urban spaces employ differentiated spatial representations at various scales, providing important references and insights for garden research (Table 10). At the architectural and interior scales, VGA is widely used to reveal local visibility and patterns of occupancy. In office settings, employee interaction frequency in high-integration areas is 2–3 times higher than in low-integration areas[55]. In school buildings, the integration of primary corridors is 30%–50% higher than that of secondary corridors, exerting a significant influence on students' route choice and space use[56]. In hospital settings, the visibility of wards and corridors is directly related to staff rounding efficiency and patient accessibility[57]. In museum spaces, the visual integration of galleries is highly correlated with visitors' tour routes and dwell hotspots[58]. At the neighborhood and city scales, studies commonly employ segment analysis to explain traffic flows and block connectivity. The correlation between global street integration and pedestrian movement patterns can reach R² ≈ 0.6–0.7[59]. Further studies indicate that traffic volumes on highly connected streets are 40%–60% higher than on low-connectivity streets[60].

In summary, empirical studies across scales consistently show that areas with high visibility and high integration are key to explaining human aggregation and movement behavior. This not only reinforces the methodological validity of garden research as a "small-scale urban space," but also suggests the need for cross-scale comparisons to expand the generalizability and explanatory power of garden studies.”

We also added **Table 10. “Comparison of spatial representations and behavioral outcomes across scales.”** to present a concise, side-by-side summary (scale → representation used → key metrics → typical behavioral outcomes → references).

**3.Comment:** Instead, the authors demonstrate that the studies themselves have only had surface-level examination. This is evident from examples such as:

a. attributing analysis to Gomaa et al. (32) using "Local integration" whereas the paper in question only mentions the metric in passing in the introduction,

**Response:** Thank you for pointing this out. We have corrected our reading of **Gomaa et al.** and revised the manuscript accordingly:

a. **Table update.** In **Table 4. “Characteristics of the studies”** we have revised the entry for **Gomaa et al.** to accurately reflect the methods reported in the paper. The table now specifies the representation and the metrics actually used (e.g., **step depth from the main entrance, choice, connectivity**, and **integration** where applicable), and removes any implication that the study conducted a full “local-integration” analysis when it was only mentioned in passing.
b. In **Section 3.4 “The influence of garden spatial configuration on tourist behavior,”** Specific content added: “Gomaa et al. [33] further found that **step depth from the main entrance** effectively reflects differences in visitor accessibility within the park, and that metrics such as **integration** and **choice** reveal the effects of spatial configuration on visitors’ route choice and perceived accessibility.”

**4.Comment:** referring to "depth" as a metric, which is unusable on its own and requires a location descriptor (as in Mohamed et al. (2024)[29] actually referring to it as "Step depth from the main entrance")

**Response:** Thank you for pointing this out. We agree that **“depth” is not interpretable on its own and requires a location descriptor**. Accordingly, we have revised **Table 7. “The influence of space syntax metrics on tourist behavior.”** as follows:

- The metric column now specifies **“Step depth (from the main entrance)”** (and where relevant, “from other nodes”), rather than using an unqualified “depth.”
- We added a concise definition and interpretation: average step distance to the main entrance or other nodes; values lower than the system mean indicate centrality, higher values indicate peripherality.
- Behavioral implication is clarified: high step depth → poor accessibility/avoidance; low step depth → high permeability.
- The Author (Year) column lists representative sources (e.g., Gomaa et al., 2024; Huang & Lee, 2023; Traunmüller et al., 2023) that explicitly operationalize step depth with a location descriptor.

**5.Comment:** having both "connectivity" (Wu et al. (2025) [30]) and "isovist area" (Yu et al. (2016) [31]) as different metrics where they are practically the same because the two studies use VGA,

**Response:** Thank you for raising this point. We agree that both measures can be produced within a visibility-based framework, but they are conceptually and computationally distinct.

- Following *Hillier & Hanson (1984), connectivity* in space syntax was originally defined on axial/convex graphs as a topological adjacency count.
- In VGA (Turner et al., 2001), VGA-connectivity is computed as the number of intervisible nodes on a sampled visibility grid—still a discrete, graph-based measure.
- By contrast, Isovist Area (Benedikt, 1979) is the continuous visible area from a viewpoint (a metric property of the isovist polygon), not a node-degree count.

Therefore, while both may correlate when interpreting visual accessibility, they are not “practically the same” and capture different aspects of visibility (discrete intervisibility vs. continuous visible extent).

To prevent confusion, we have updated **Table 7. “The influence of space syntax metrics on tourist behavior.”** and added a note: “Both measures can be derived from visibility graph analysis (VGA), but they differ conceptually and computationally. **Connectivity** measures intervisible points, while **Isovist Area** measures continuous visible space.”

**References:**
Hillier, B., & Hanson, J. (1984). *The Social Logic of Space*. Cambridge Univ. Press.
Turner, A., Doxa, M., O’Sullivan, D., & Penn, A. (2001). From isovists to visibility graphs. *Environment and Planning B*, 28(1), 103–121.
Benedikt, M. L. (1979). To take hold of space: Isovists and isovist fields. *Environment and Planning B*, 6(1), 47–65.

**6.Comment:**mentioning "extended metrics" in Tables 6 and 7, attributing it to different papers but never explaining what those metrics are

**Response:** Thank you for highlighting this gap.We have now defined and exemplified **extended metrics** in a new subsection **“3.3.4. Isovist Area, Visual Integration and Extended Metrics.”**

Specific content added: “Extended metrics refer to composite measures derived from space syntax core metrics (integration, connectivity, depth) in combination with other data or analytical tools. Common forms include combining space syntax with heatmaps, GPS trajectories, or questionnaire results. By integrating space syntax metrics with behavioral or perceptual data, extended metrics can systematically reveal associations between spatial characteristics and outcomes such as visitor clustering, satisfaction, and revisit intention[29]. They overcome the limitations of single metrics and, through multi-dimensional data integration, enhance the explanatory and predictive power for the relationship between garden spatial structure and tourist behavior. Huang and Lee.[28] examined the integration of Baidu heatmaps with space syntax and found a statistically significant correlation between integration and visitor clustering intensity.”

In addition, in the note to **Table 7: “The influence of space syntax metrics on tourist behavior,”** we explicitly state that “extended metrics are not new space syntax indices but analyses paired with external data,” and we list representative forms (e.g., GPS trajectories, heatmaps, and questionnaire-based measures).

**7.Comment:** completely ignoring other important metrics such as control found in Mohamed et al. (2024)[29] and choice found in Wu et al (2025) [30].

**Response:** Thank you for pointing this out. We have addressed the omission of control and choice in two ways:

New subsection added. In **Section 3.3.2 “Control value and Choice,”**

Specific content added: “Mohamed et al. [30] indicate that the **control value** measures the relative control of a spatial node over its adjacent nodes within a spatial network, reflecting the functional mechanism of the focal spatial unit in the local spatial structure and serving to assess its effects on surrounding spatial units…. Higher choice values indicate that the spatial unit plays a central role in route selection within the overall network [45].”

Table updated. We have updated **Table 7. “The influence of space syntax metrics on tourist behavior.”** to explicitly include **control** and **choice**，and cited representative studies

**8.Comment:** This shallow approach has also prevented the authors from drawing larger conclusions in their discussion such as how and, more importantly, why some metrics affect tourist behaviour. For example the authors do identify that Integration is an important metric but do not go further to try to identify why it might correlate with tourist path selection. Is it perhaps a strategy of people who don't know a space (tourists) to traverse that space on what looks to them as the most obvious path? Is it that this is the result of exploratory behaviour as in other cases (say galleries)? The authors instead avoid the larger critical reflections (which would be fitting for a such a review) and choose to recite the various results from the studies highlighting the limitations (which seem to also come from the studies).

**Response:** Thank you for this important comment.

we have added：**Table 7. “The influence of space syntax metrics on tourist behavior.”** This new table links each metric to its operational range/criteria and explicit behavioural effects (e.g., path choice, dwell, accessibility), so the manuscript now explains why metrics such as integration, connectivity, choice, and step depth (from the main entrance) correlate with tourist route selection and movement patterns.

we have added：Section 4.3. “Structure–Perception–Behavior (SPB) framework,” with Fig 6. “Structure–Perception–Behavior (SPB) framework.” has addressed deeper-level reflections

Othe notes:

The referencing is inconsistent to the point where it creates confusion:

1. One of the main studies "Zhang et al. (2020)" referenced as 15 has a broken DOI and either the wrong title or the wrong authors. The title matches authors Zhang T., Lian Z., and Xu Y and the DOI <https://doi.org/10.1080/01426397.2020.1730775.>

**Response:** Zhang, T., Lian, Z., & Xu, Y. (2020). Combining GPS and space syntax analysis to improve understanding of visitor temporal–spatial behaviour: A case study of the Lion Grove in China. *Landscape Research*, *45*(4), 534-546.<https://doi.org/10.1080/01426397.2020.1730775>

2. It is unclear what "extended metrics" are, they are attributed to either Lee, K. (2021) in Table 6 or Huang and Lee (2023) in Table 7

**Response:** Thank you for pointing out the ambiguity around “extended metrics.” We have clarified both the definition and the attribution as follows:

Definition added (Section 3.3.4. “Isovist Area, Visual Integration and Extended Metrics”).

Specific content added: “Extended metrics refer to composite measures derived from space syntax core metrics (e.g., integration, connectivity, depth) in combination with other data or analytical tools. Typical forms include coupling space syntax with heatmaps, GPS trajectories, or questionnaire-based perceptual scores… Huang & Lee [28] integrate Baidu heatmaps with space syntax and report a statistically significant correlation between integration and visitor clustering intensity.”

We added Table 7. “The influence of space syntax metrics on tourist behavior.” and explicitly labeled which entries fall under extended metrics.

In Table 7, Zhang et al. (2020) corresponds to space syntax × GPS; Huang & Lee (2023) corresponds to space syntax × heatmap. Both are treated as extended metrics.

**Reviewer #4:**

**1.Comment:** The biggest issue with the paper is the unclarity of its questions. The authors fail to clearly highlight the specific research questions they themselves aim to sequentially address in this study – apart from the questions that were raised by the articles of the literature review. All of which confuses the reader. As the authors emphasized that their main research interest was 'Examining the relationship between garden spaces and touristic behavior,' they posed two questions. While RQ1 is not clear in its academic intent, and could therefore be adjusted, RQ2 is more relevant but would benefit from being developed into several operational sub-questions (among which the modified/altered RQ1 could be incorporated). This lack of clarity in the formulation of the research questions directly affects the overall rigor of the study. In its current form, the paper does not adequately address the research operational questions, leaving the reader with a conclusion that offers little specificity regarding results relevant to the central focus. The final sections continue to reference existing literature rather than engaging with the study’s own findings. A stronger approach would have been to adopt a singular overarching focus (examining the relationship between garden spaces and touristic behavior), develop several operational sub-questions, rigorously investigate each of them, and then provide a conclusive answer supported by empirical evidence/finding. Although the authors have made considerable effort and generated the findings needed, the knowledge that they developed is not presented in a structured or clear way; important findings are thrown as familiar statements, and the headings and the sub-headings of the sections do not always hint to the link between the question inquired and the content presented. Based on a review of the current content of their study, the authors should formulate three to four operational questions and restructure the study to present clear, definitive answers.
In general, the authors need to revise the text, develop a multi-layered discussion, and clearly highlight their contributions at three levels: (1) data collection and PRISMA findings, (2) the application of space syntax metrics and how these metrics are used and analyzed to reveal specific configurational–behavioral experiences - in addition to the other supporting theories if any, and (3) the interpretations derived from the multi-layered study and the cumulative insights of the ten reviewed papers.

**Response:** Thank you for highlighting the lack of clear, sequential research questions and layered discussion. We have revised the manuscript as follows:

1) Section “1. Introduction” — Research questions added

We explicitly state the study questions:

RQ1: From 2015 to 2025, what distribution and evolutionary patterns are exhibited in the research characteristics and publication features of space-syntax-based studies on ''garden spatial configuration and tourist behavior''?

RQ2a: Which core metrics of space syntax were employed in the included studies? What are the definitions and computational formulas of these metrics?

RQ2b: How do different metric characteristics and spatial representations in space syntax (VGA, Segment, Isovist, Convex) influence tourist behavioral outcomes?

RQ2c: Which features have existing studies used to reveal the mediating role of tourist perception in the relationship between spatial configuration and behavioral outcomes?

2) Section “2.3. Study selection” — PRISMA process detailed

We provide full PRISMA numbers and decision rules and updated the figure:

“In accordance with PRISMA 2020, 1,040 records were retrieved from four databases; after removing 119 duplicates, 921 proceeded to title/abstract screening: 174 were excluded for timeframe mismatch, 77 were books, and 541 were unrelated to the topic. A total of 129 full texts were obtained and assessed: based on PICOS, 7 reviews/non-empirical studies, 11 without space syntax, 9 without tourists/visitors, 83 outside garden/park/woodland contexts, and 1 abstract-only/no full text were excluded. Ultimately, 18 studies were included (Fig. 2). In cases of disagreement during the screening process, a third expert was consulted to assist in reaching a final consensus.” Figure label: **“Fig 2. PRISMA flow diagram.”**

3) Section “3.4. The influence of garden spatial configuration on tourist behavior” — Mechanism-oriented mapping of metrics to behavior

We added a synthesis table linking each metric to its operational range/criteria and concrete behavioral effects:

**“Table 7. The influence of space syntax metrics on tourist behavior.”**
This section addresses **how** space-syntax metrics (e.g., integration, connectivity, choice, step depth from the main entrance; visual integration, isovist area) explain route choice, dwell, and accessibility.

4) Section “4.3. Structure–Perception–Behavior (SPB) framework” & “4.4. Cross-scale methodological implications for garden spatial research” We provide the review’s higher-level reflection by introducing a mechanism pathway and cross-scale perspective:

4.3.Structure–Perception–Behavior (SPB) framework, Specific content added: “Based on a systematic review of the relevant literature, this study proposes the Structure–Perception–Behavior (SPB) framework (Fig. 6). ….The significance of this framework lies in integrating previously fragmented empirical findings into a testable theoretical model, thereby providing a basis for further verification and refinement in future quantitative studies, and advancing research on the relationship between garden spatial configuration and tourist behavior toward greater systematization and theorization.” We have added: “Fig 6. Structure–Perception–Behavior (SPB) framework.”

4.4.Cross-scale methodological implications for garden spatial research,Specific content added: “This review centers on garden spatial configuration，emphasizing gardens as a typical "small-scale urban space." ……..This not only reinforces the methodological validity of garden research as a "small-scale urban space," but also suggests the need for cross-scale comparisons to expand the generalizability and explanatory power of garden studies.” We have added Table 10, “Comparison of spatial representations and behavioral outcomes across scales.”

**2.Comment:** The methodology outlining how the collected data is investigated, interpreted, and how each layer of the analysis and research questions is addressed needs to be presented more clearly, both in text and through a flow chart. While the authors included visual graphs and tables related to the literature review and its summary. However, in the sections /levels where their own input is most important and potentially influential, this impact is minimized. Summaries concluded section-paragraphs, interpretations, and visualizations should be used more effectively to emphasize their contribution and to clearly demonstrate the methodology applied in screening and analyzing (the content of) the selected papers, which is critical. A chart detailing this methodology is expected to be incorporated prior to the presentation of the results. Furthermore, the Methods section unnecessarily expands on subsections 2.2 (Criteria) and 2.4 (Quality Assessment), which distracts the flow of the main core topic. It could be more effectively reduced and summarized in a simple paragraph and a table. It should also be clarified that, while many studies employ similar large databases, the relatively small number of papers reviewed in this specific topic limits the extent to which statistical findings alone can be relied upon. This makes a deeper level of analysis and interpretation in this study particularly important.

**Response:** Thank you for the suggestion. We have clarified the methodology with a dedicated flow chart placed at the end of the **Introduction**, titled **“Fig 1. Flow diagram for literature review.”**

We have streamlined the Methods by **merging Sections 2.2 (Criteria) and 2.4 (Quality Assessment) into “2.2 Criteria and quality assessment.”** The subsection is now presented as a concise paragraph plus a table.

Specific content added:

“This study employed the PICOS framework for literature screening [24]; the inclusion criteria simultaneously satisfied the following conditions (Table 2). Although the search covered major databases, the number of studies ultimately included was relatively small; therefore, this study does not rely solely on statistical frequencies but adopts an interpretive synthesis, emphasizing the correspondence between ‘metrics–representations–behavior’ and the elucidation of the ‘structure–perception–behavior’ mechanism. This study employed the Crowe Critical Appraisal Tool (CCAT) for quality appraisal [25]. Developed by Lynne Crowe, the tool is applicable to quantitative, qualitative, and mixed-methods studies and provides a standardized evaluation framework [26]. The assessment comprises the following dimensions: introduction, background, methods, abstract, data collection, ethics, results, and discussion. Each dimension is scored from 1 to 5, with no half points [27] (S2 Table).”

**3.Comment:**While Table 4. - which appears in section 3.1., provides a comprehensive summary and introduces several socio-perceptual and phenomenological tools/theories, it would be useful to include a paragraph(s) briefly describing the other tools (beyond space syntax) that are mentioned. In other words, authors need to present a brief explanation to the “theories” that appear in the column of “theory” Table 4., and to clarify how these relate, or not relate, to Space Syntax, and consequently the title of this study. (Examples TG, CL, NRT, IL, TB,….. and why they are included). In addition, the meaning of 'Visitor involvement level' in Table 4 is unclear; its relevance to the investigation should be clarified; otherwise, it could be omitted.

**Response:** Thank you for the suggestion. To avoid a methodological misreading that would equate “socio-perceptual/phenomenological tools & theories” with space syntax metrics, we removed the “theory” column from Table 4 and kept the table focused on items directly tied to space syntax and our coding scheme. Because the definition of “Visitor involvement level” varies across studies (most papers do not provide a comparable operational definition or a consistent quantification) and it does not align fully with our coding dimensions, we deleted this column as well. We updated the column structure and footnotes of Table 4, reorganized Table 4 (“Characteristics of the studies”)

**4.Comment:**Table 6. in Section 3.3 could be expanded by adding images of the layouts of the 10 cases studied with certain space syntax and visibility maps (with clear references). The visuals of the space syntax metrics as reflected in graphs usually help understanding the findings and help in understanding the context.

**Response:** Thank you for the helpful suggestion. We have revised the presentation accordingly: the plan layouts of the 18 included cases, together with their corresponding space syntax/visibility maps (e.g., VGA, Segment, Isovist/Axial) and clear references, have been compiled into Table 4. “Characteristics of the studies.” This consolidated view improves contextual understanding of each case and makes the reported metrics and findings easier to interpret.

**5.Comment:**To improve the readability of Table 7 which appears in Section 3.4, it would be helpful to add a column entitled “Space Syntax Metrics” to clarify the metrics used in each of the studies listed. Alternatively, Tables 6 and 7 could be combined into a single table.

**Response:** Thank you for the suggestion. We have merged Tables 6 and 7 into a single table titled “Table 7. The influence of space syntax metrics on tourist behavior.” To improve readability, we also added a new column, “Space Syntax Metrics,” which explicitly lists the metric(s) used in each study. The consolidated table now presents, for every study, the metric(s), range/criteria, behavioral effects, and references.

**6.Comment:** Section 3.5 is an important part of the paper as it highlights the distinction between the impact of garden spatial configuration and perceptual values such as aesthetic preferences, cultural cognition, and sense of safety. The authors should emphasize this section more clearly to underline their contribution. Including a table that summarizes these issues would also strengthen the presentation.

**Response:** Thank you for underscoring the importance of Section 3.5. We have substantially rewritten this section and added a summary table: Section 3.5 “Tourist perception as a mediating effect.” Rewritten content: “Among the 18 reviewed studies, tourist behavior is not entirely determined by the spatial structure of the garden itself. Tourists' subjective perceptions are an indirect factor influencing behavioral outcomes such as dwell time, movement speed, and social interaction, and are analyzed via perceptual variables including aesthetic preferences, cultural cognition, safety, and restoration[53] (Table 8). Zhang et al.[29] pointed out that the spectatorship of garden nodes engenders a "view-at-every-step" aesthetic perception; the revisitation rate increases significantly and average walking speed decreases, and when directional perception is clearer, route choice becomes more stable. Yu et al.[39] further support this view: visual perceptions such as coherence, openness, and legibility can significantly predict dwell density, evoke emotional experiences including immersion, mystery, safety, and imageability, and promote prolonged dwelling and contextual immersion. Chen and Yang.[36] confirmed the mediating role of cultural cognition; narrative elements such as plaque inscriptions, couplets, and poetry-painting serve as carriers that more readily evoke visitors' understanding and experience of the "garden within a garden," thereby reinforcing visual guidance and deepening overall spatial memory. Tourists' restorative perception directly affects dwelling behavior，Saadativaghar et al.[35] showed that restorative perception is stably associated with spatial configuration and can predict dwell duration and intention, further indicating its key mediating role in linking space and behavior. Meanwhile, Mohammadi and Ujang.[37] indicate that landmarks and activity nodes help enhance perceived social safety, thereby promoting social interaction and dwelling behavior.

In summary, tourists' perceptions—encompassing aesthetics, culture, safety, and restoration—mediate the relationship between garden spatial configuration and behavioral performance. By modulating processes such as directional cognition, route choice, dwell duration, and social interaction, these factors indirectly shape behavioral patterns. This lays the groundwork for a subsequent ''Structure–Perception–Behavior'' framework and offers a perspective for explaining behavioral differences across cultural and environmental contexts. ”

**Table added:** **“Table 8. The impact of tourist perception on behavioral outcomes.”** This table synthesizes the perception variables (aesthetic preference, cultural cognition, safety, restoration), their links to spatial features, and the corresponding behavioral effects (e.g., dwell, speed, route stability, social interaction) with citations.

**7.Comment:** The presentation of the text in Section 4.1 gives the impression of abundance and unnecessary repetition. The authors should clarify the distinctions between the metrics, their analytical value, and the interpretive insights that emerge when different space syntax indicators are combined. Since the power of integrating tools is central to this section and represents the authors’ key contribution, it should not be downplayed by the inclusion of repeated content. Reference to the 10 studied papers could help (Optional).

**Response:** Thank you for the helpful guidance. We have addressed the concern about Section 4.1 as follows: We removed repetitive statements from 4.1 “Characteristics of space syntax indicators,” avoiding study-by-study repetition. To prevent redundancy, we moved and consolidated the explanations of each metric into **3.3 “Characteristics of space syntax indicators” (3.3.1–3.3.4)**, providing for each metric a brief definition, analytical role/value, and key interpretive notes.

**8.Comment:** The authors are advised to include a table at the end of Section 4.2, followed by an interpretive paragraph.

**Response:** Thank you for the suggestion. In **Section 4.2**, we have added a new table and a concise interpretive paragraph: **Table added:** **“Table 8. The impact of tourist perception on behavioral outcomes.”** The table synthesizes perception variables (aesthetic preference, cultural cognition, safety, restoration), their links to spatial features, and corresponding behavioral effects (e.g., dwell density, route stability, social interaction), with citations. Interpretive paragraph (appended after the table): “In summary, tourists' perceptions—encompassing aesthetics, culture, safety, and restoration—mediate the relationship between garden spatial configuration and behavioral performance. By modulating processes such as directional cognition, route choice, dwell duration, and social interaction, these factors indirectly shape behavioral patterns. This lays the groundwork for a subsequent ''Structure–Perception–Behavior'' framework and offers a perspective for explaining behavioral differences across cultural and environmental contexts. ”

**9.Comment:** Section 4.4 would benefit from a concluding paragraph that summarizes the findings and highlights the authors’ interpretations, thereby underscoring their own voice and contribution.

**Response:** Thank you for this suggestion. To underscore our own interpretations and contribution, we have augmented the Discussion with two focused, concluding components:

Section 4.3 “Structure–Perception–Behavior (SPB) framework.”

Specific content added: “Based on a systematic review of the relevant literature, this study proposes the Structure–Perception–Behavior (SPB) framework (Fig. 6). …..The significance of this framework lies in integrating previously fragmented empirical findings into a testable theoretical model, thereby providing a basis for further verification and refinement in future quantitative studies, and advancing research on the relationship between garden spatial configuration and tourist behavior toward greater systematization and theorization.”

Section 4.4 “Cross-scale methodological implications for garden spatial research.”

Specific content added: “This review centers on garden spatial configuration，emphasizing gardens as a typical "small-scale urban space." …..This not only reinforces the methodological validity of garden research as a "small-scale urban space," but also suggests the need for cross-scale comparisons to expand the generalizability and explanatory power of garden studies.”

**10.Comment:** The conclusions should directly address the research questions and provide clear answers at both the local and global levels.

**Response:** Thank you for the guidance. We have rewritten Section 6 (Conclusions) so that it directly answers the research questions and provides takeaways at both the local and global levels: The revised conclusion reads:

“6.Conclusions

This systematic review explores the relationship between garden spatial configuration and tourist behavior, offering meaningful insights into the multiple factors influencing behavioral patterns. The results indicate an overall upward trend over time and across regions, with research subjects primarily focusing on traditional gardens in East Asia. Space syntax has become a key tool for studies on gardens and tourist behavior. Regarding core metrics, integration, connectivity, depth, as well as isovist area and visual integration, are widely used and can effectively predict visitor movement paths, hotspot areas, and spatial preferences.Different spatial representations (VGA, segment, isovist, convex) respectively emphasize the differentiated effects on visual connectivity, route choice, and perceptual behavior. In addition, variables such as aesthetic preference, cultural cognition, and sense of safety play important mediating roles within the spatial structure, forming a “structure–perception–behavior” pathway, which integrates key findings of existing studies at the local level and, at the overall level, provides a theoretical framework and methodological support for understanding the multiple impacts of garden space on tourist behavior. This review not only demonstrates the applicability of space syntax to garden planning and tourist behavior research, but also provides directions for future studies to develop empirical models to test mediating mechanisms, thereby enhancing the breadth and theoretical depth of the research. ”

Other issues:

One,

The following paper that you referred to in your study is an excellent example, particularly in how it demonstrates the alignment between paragraphs and figures (see, for instance, Figures 3 and 4): Lee, J.H.; Ostwald, M.J.; Zhou, L. (2023). Socio-Spatial Experience in Space Syntax Research: A PRISMA-Compliant Review. Buildings, 13(3), 644. <https://doi.org/10.3390/buildings13030644>

**Response:** Thank you for the pointer. We consulted **Lee, Ostwald & Zhou (2023)** as a model for aligning narrative and visuals and, drawing on our study’s scope, created a new schematic titled **“Fig 5. Space Syntax and Tourist Outcomes.”**

Two,

A note to look at: At the 4th International Space Syntax Symposium in 2003, M. Abrioux presented an article entitled 'Body, eye and imagination: A meditation on the dynamics of space in French and English gardening.' The paper applied space syntax to garden analysis, emphasizing the perceptual and imaginative dimensions of spatial experience in French and English garden design. Although not a comprehensive study of gardens, it represents a key contribution in that year by extending space syntax concepts into the context of landscape architecture and highlighting how garden design can shape visitors’ perception and experience of space. The research likely focused on the subtle, qualitative aspects of spatial design rather than just quantitative metrics, which are more common in other space syntax applications. It might help to add this paper to your references for its direct relevance to the chronological literature review. Following are the details of the reference. Abrioux, M. (2003). Body, eye and imagination: A meditation on the dynamics of space in French and English gardening. Paper presented at the 4th International Space Syntax Symposium, London.

**Response:** Thank you for the recommendation. We have cited the suggested work in **Section 1. Introduction** and added the following sentence:

“Space syntax is linked to the perceptual and imaginative dimensions of gardens; using English and French horticulture as examples, Abrioux [15] discusses how ‘body–vision–imagination’ shapes spatial experience, providing a conceptual precursor for subsequent garden research that employs perception as a mediating factor.”
