# Supplementary material for: The influence of garden spatial configuration on tourist behavior: A systematic review based on Space Syntax
Source: PLoS One. 2026 Jan 2;21(1):e0339994. doi: 10.1371/journal.pone.0339994 (PMC12758741; doi:10.1371/journal.pone.0339994)
Supplement: Attachment — Submitted filename: Response_to_Reviewers_auresp_2.docx [file pone.0339994.s007.docx]

Response to Reviewers' Comments

Title: The Influence of Garden Spatial Configuration on Tourist Behavior: A Systematic Review Based on Space Syntax

Manuscript Number : PONE-D-25-34363R1

We sincerely thank the academic editor and reviewers for their valuable feedback. We have addressed all comments and revised the manuscript accordingly. Our detailed responses are provided below.

**Journal Requirements:**

1. If the reviewer comments include a recommendation to cite specific previously published works, please review and evaluate these publications to determine whether they are relevant and should be cited. There is no requirement to cite these works unless the editor has indicated otherwise.

**Response:** Thank you for the guidance. In line with the journal’s requirement, we carefully reviewed the publications mentioned by the reviewer. Following an independent relevance check, we found that these works are not essential to the specific focus of our review (garden-scale spatial configuration and tourist behavior using space syntax) and would introduce overlap without adding substantive value. Accordingly, we have removed those citations in the revision.

2. Please review your reference list to ensure that it is complete and correct. If you have cited papers that have been retracted, please include the rationale for doing so in the manuscript text, or remove these references and replace them with relevant current references. Any changes to the reference list should be mentioned in the rebuttal letter that accompanies your revised manuscript. If you need to cite a retracted article, indicate the article’s retracted status in the References list and also include a citation and full reference for the retraction notice.

**Response:** We have rechecked all references one by one to ensure completeness, accuracy, and retraction status. To the best of our knowledge at this revision stage, none of the cited works have been retracted. If any item is later found to be retracted, we will replace it with the most current and relevant source in accordance with the journal’s policy.

**_____________________________________________________________________**

**Reviewer #1: (No Response)**

**Reviewer #2:**

**1.Suggestions for Minor Enhancement:**

**(1)** Clarify whether the SPB framework is a synthesis from the literature or a proposed theoretical model.

**Response:** Thank you for this helpful suggestion. We now explicitly clarify the provenance of the SPB framework in **Section 4.3.Structure-Perception-Behavior (SPB) framework** : it is a theory-informed synthesis from the literature, not a wholly new theory. Building on Mehrabian & Russell’s Stimulus–Organism–Response (SOR) model and Rapoport’s Culture–Environment–Behavior (CEB) framework, we articulate the mediating mechanism as X (spatial structure via space-syntax metrics) → M (tourist perception: aesthetics, legibility, cultural cognition, safety, restoration) → Y (behavioral outcomes: route choice, dwell time, social interaction), and construct the resulting Structure–Perception–Behavior (SPB) framework. The revised wording is: “Based on a systematic review of the relevant literature, and drawing on Mehrabian and Russell’s SOR model and Rapoport’s CEB explanatory framework, this study clarifies the “X → M → Y” mediating mechanism and constructs a “structure–perception–behavior” (SPB) framework.”

**(2)** Provide a concise explanation of the correlation between behavioural outcomes (e.g., cognitive ease, legibility) and metrics such as integration and choice.

**Response:** Thank you for the suggestion. We have added a concise explanation **in Section 4.3 “Structure–Perception–Behavior (SPB) framework.**” The new text clarifies how behavioural outcomes (e.g., cognitive ease and legibility) relate to key space-syntax metrics:

“Spaces with high integration typically exhibit greater visual centrality and spatial accessibility. Their clear orientation and easily recognizable routes enhance the spatial configuration's identifiability and legibility. Accordingly, the more recognizable and legible a space is, the higher its integration tends to be. In addition, high Choice increases the likelihood that a path functions as a primary route, and the formation of primary routes, in turn, reinforces high Choice. Through this mutual influence, high-choice main routes often become centers of human activity and aggregation and are more prone to high pedestrian flow and congestion. Consequently, Choice is positively associated with behavioral outcomes: the higher the Choice value, the greater the probability that a path will be traversed, thereby shaping tourists' route preference decisions.”

**(3)**  Include a brief commentary on the cultural distinctions between Western and Eastern garden typologies.

**Response:** Thank you for the helpful suggestion. We have added a brief commentary on cultural distinctions between Western and Eastern garden typologies in Section 1. Introduction. The new paragraph reads:

“Across different civilizational lineages, the evolutionary trajectories and spatial connotations of garden types differ markedly. For instance, Eastern gardens, influenced by Confucian, Taoist, and Buddhist philosophies, emphasize the creation of spaces that embody the "unity of heaven and humanity," personal cultivation, and transcendent mental states. Within this framework, Chinese imperial gardens, shaped by ritual systems and imperial discourse, emphasize grand layouts and central axis order, symbolizing political power. Private gardens, however, favored the "microcosmic" landscape aesthetic, emphasizing the literati's appreciation of shifting vistas and self-cultivation through "scenes that change with every step."Japanese gardens, inheriting early Chinese Buddhist traditions, ultimately evolved under the influence of Zen and the tea ceremony to use stones, sand, and moss as primary elements, creating wabi-sabi aesthetics and meditative spaces. In contrast to the Eastern pursuit of natural beauty, Western gardens emphasize the unity of religion and power through converging axes and waterways. Examples include Renaissance gardens that express "rational domination over nature" through geometric order, and Baroque gardens that reinforce monarchical authority through spatial hierarchy. Within contemporary urban contexts, gardens, whether rooted in Eastern traditions or Western lineages, have become spatial vessels for recreation, sightseeing, and social interaction for both residents and visitors. ”

**(4)** The final round of English language and formatting refinement will be conducted.

**Response:** We conducted in-depth revisions to key sections—the Abstract, Introduction, Conclusions, and Limitations—and applied targeted language and formatting edits to the remaining content to improve clarity and coherence. No substantive changes were made to the study design, data, or results.

**Reviewer #3:**

**1.**In response to Reviewer #3, 5. Comment Response: I mentioned Connectivity and Isovist Area as the same exactly _because both studies use VGA_ in which case they are the indeed practically the same. The original Hillier Connectivity refers to axial connections (essentially the number of junctions of a street), but in VGA Connectivity (or Visual Connectivity if you'd rather make this clearer) refers to the number of cells that are visible from a cell. This is calculated by creating a 360-degree isovist from each cell and looking at the number of cells that fall within the isovist polygon. The larger the area of the isovist polygon (what Isovist Area measures), the more the cells; and the smaller the cells, the more Visual Connectivity will approach Isovist Area. There is a finer point about intervisibility, but the reality of the VGA grid cell construct is that there are no non-reciprocal relationships (i.e. every cell that cell A can "see" can also "see" A). This is exactly because we use 360-degree isovists and because there are no studies (as far as I'm aware) that actually look at VGA with one-way visibility materials (though even in that case the practical - what the code outputs - result will once again be the same). If you'd like to dig deeper into the details of VGA metric calculation look at Koutsolampros (2021) section 4.3.1 on page 124, or Koutsolampros et al. (2019).

**Response:** Thank you for the clear explanation and the references. We sincerely appreciate your professional guidance—this suggestion has significantly improved the precision of the manuscript’s terminology.

**2.** In section 3.1 it is not immediately clear that you mean that there are identically named metrics across representations. I would rephrase otherwise it seems that Integration and Connectivity are somehow identically named between them.

**Response:** Thank you for flagging the potential ambiguity. To avoid the impression that metrics are identically named across different representations, we have revised Section 3.1 (“Research characteristics”). The paragraph now reads:

“This study provides a systematic synthesis of the 18 included studies (Table 4). The general characteristics were summarized across the following dimensions: country of publication, study location, spatial analysis, and space syntax modeling methods, core space syntax metrics (integration, connectivity, choice, control), radius or weighting settings, and reported outcomes. Specifically, spatial analysis and space syntax modeling methods comprised two categories: (1) visualization- or statistics-based analyses, including kernel density estimation (KDE), heatmaps, and experiential maps; and (2) space syntax modeling methods, including axial maps, visibility graph analysis (VGA), segment analysis, and isovist-based models. Notably, axial, segment, and VGA analyses emphasize the global network structure or connectivity, whereas the isovist analysis focuses on local field-of-view visibility”.

**3.** While table 4 is certainly welcome it seems that there has not been enough attention to detail:

**3.a.** For Huang & Lee (2023), "Kernel density estimation" and "heatmap" are not spatial representations.

**Response:** Thank you for the reviewer’s correction. We agree that Kernel Density Estimation (KDE) and heatmaps are **not** spatial representation/modeling methods. We have corrected **Table 4: Characteristics of the studies**, reclassifying them under **“Visualization and statistical analysis”** rather than “Spatial representation/model,” and we have updated the table headers and footnotes accordingly and standardized the terminology throughout the manuscript.

In addition, for reasons of scholarly transparency and copyright compliance: most illustrative figures are owned by the original articles or institutions and should not be republished without explicit permission; even with attribution, such reuse may exceed fair-use limits. Therefore, we have made minor structural adjustments to **Table 4. Characteristics of the studies** and, in the table notes, direct readers to consult the original figures via the corresponding reference numbers.

**3.b.** For Chen & Yang (2025), what are "Isovist fields"? These are not explained anywhere.

**Response:** Thank you for pointing this out. Upon verification, we found that Chen & Yang (2025) should in fact be Chen & Yang (2023). This has been corrected in **Table 4. Characteristics of the studies**, and the modeling method has been explicitly identified as “Isovist (visibility model).” To avoid ambiguity, we have replaced “isovist fields” with “isovist” throughout the manuscript and added the following note in the main text and in the Table 4 notes: Isovist: the portion of space visible from a given observation point under conditions of spatial occlusion.

**3.c.** For Mohammadi & Ujang (2021), what are experiential maps? Not explained anywhere. Are they spatial representations? How?

**Response:** Thank you for the clarification request. We have clarified in the table notes that “experiential maps” are defined as: “Experiential maps: records of participants’ subjective experiences and behaviors within the site.” Accordingly, in Table 4. Characteristics of the studies, we have updated the column header to “Spatial Analysis and Modeling Methods” to avoid ambiguity.

**3.d.** For Yu et al. (2021), it seems the authors use convex, not axial, even if they mention axial in some places.

**Response:** Thank you for the helpful observation. We have corrected the entry for **Yu et al. (2021)** in **Table 4. *Characteristics of the studies***: the space-syntax representation has been updated from **“axial”** to **“convex.”**

**3.e.** For Zhang et al. (2019), you say "VGA + Depthmap", but Depthmap is just the tool for (among other things) carry out VGA.

**Response:** Thank you for the correction. To improve accuracy, we have updated **Table 4. Characteristics of the studies** by changing Zhang et al. (2019) from “VGA + Depthmap” to **VGA** only.

**3.f.** For Chen et al. (2025), you say "Isovist polygons & Parameters", why not just "Isovists", why is this different to "Isovist fields" above, and what are "Parameters" (certainly not a representation).

**Response:** Thank you for the suggestion. We have standardized the terminology in **Table 4: Characteristics of the studies**, correcting “Isovist polygons & Parameters” to **“Isovists ”**, and added a footnote definition to avoid ambiguity. Specifically: “Isovist: the visible space from a given point under conditions of spatial occlusion.”

**4.** In section 3.3 "visual" indicators are also "topological" due to the way that the grid is constructed (see comment 1 and related references). Either actually state the difference in measurement (topological/visual, metric, angular), or just actually specify that these refer to different representations (segment, convex, grid/VGA).

**Response:** Thank you for the constructive comment. We have **rewritten Section 3.3, “Characteristics of Space Syntax Indicators,”** to eliminate ambiguity and improve readability. The specific revisions are as follows:

“Space Syntax is a theoretical and methodological framework for analyzing the relationship between spatial structures and human behavior [46]. Spatial configuration metrics derived from Space Syntax modeling, such as those based on axial maps and segment maps, primarily include integration, connectivity, depth, control, mean depth, and relative asymmetry[47], these metrics focus on spatial accessibility and connectivity. Visual features mainly include visual connectivity, visual integration, visual selectivity, visible area, and visible boundary length[48], these are based on the VGA (Visual Area Gauge) in spatial syntactic modeling methods, and these metrics focus on visual accessibility and visual perception. Therefore, both theoretically possess topological properties, but there are certain differences in their spatial syntactic modeling methods. The 18 articles included in this study cover topics such as depth value, integration, control value, selectivity, connectivity, visible area, visual integration, and composite indicators. To further understand the core indicators of Space Syntax, this study integrates spatial attributes and their metric characteristics to provide a detailed elaboration on spatial structure and accessibility, local control and path flow, local connectivity, spatial intelligibility, isovist area and visual integration, as well as extended metrics.”

**5.** In 3.3.1 you are describing Topological/Visual or "Step" depth (number of steps to get somewhere). There's also metric depth and angular depth. Please specify the name (and ideally everywhere else).

**Response:** Thank you for the helpful suggestion. We have **revised Section 3.3.1 “Core Space Syntax Metrics”,** The specific revisions are as follows:

“Within the framework of Space Syntax theory, depth is used to analyze the topological, metric, and angular relationships between spatial units, and it is generally categorized into three types: Step Depth, Metric Depth, and Angular Depth. Step Depth is calculated based on the number of steps along a path and reflects the hierarchical relationships within the overall structure of spatial units; Metric Depth is computed using the geometric length of space and emphasizes the actual physical distance and walking cost in space; Angular Depth is calculated mainly based on changes in turning angles along the path and emphasizes people's perception of the number of turns and the magnitude of turning angles[49]. This study uses Step Depth analysis to investigate historical gardens in which surveying accuracy is limited but spatial progression is emphasized.”

**6.** In 3.3 generally; I don't understand why the grouping of metrics in this way (and it's not explained). Why "Control value and Choice"? Why does Control have "value" next to it and Choice does not? Why are Connectivity and Intelligibility in the same section? Please revise and group the metrics in a more meaningful way.

**Response:** Thank you for the reviewer’s suggestion. We have **reorganized Section 3.3, “Characteristics of space syntax indicators,”** to strengthen the grouping logic and avoid ambiguity, corrected **“Control value” to “Control,”** and, in **3.3.1 Core Space Syntax Metrics,** added **“Table 6. Characteristics of Core Space Syntax Metrics.”**

**7.** In 3.3.3, formula (6) doesn't mean anything. Intelligibility comes from using a specific statistical process - please explain it and specify the formula.

**Response:** Thank you for pointing this out. We have removed the previous expression and inserted the revised formula in **Table 6: Characteristics of Core Space Syntax Metric.**

**8.** In 3.3.4, it is a strange statement that "visual integration" is good for lingering and photography given that areas with high integration tend to be intermediate central pathways (correctly assumed to be generating social interaction). If this is from the authors then specify the exact page otherwise explain.

**Response:** Thank you for pointing this out. After re-structuring Section 3.3, the former **3.3.4** is now **3.3.2**, and we have rewritten the relevant passage to avoid the misleading claim. The revised text reads:

“Higher visual integration indicates greater spatial guidance and attractiveness. In garden environments, spaces with high visual integration are generally located in areas with strong intersect visibility, meaning that the view is less obstructed and the field of vision is open, which is more conducive to promoting social interaction and the flow of people. ”

**9.** In 3.3.4 (again), the "Common forms" mentioned are of combinations, not of metrics, making this harder to understand. What kind of metrics come out of the combinations with GPS trajectories? Also, questionnaire results are usually output or "dependent" metrics, so I wouldn't include them as metrics that may influence human behaviour or perception.

**Response:** Thank you for pointing this out. After re-structuring Section 3.3, the former **3.3.4** is now **3.3.2**, and we have rewritten the relevant passage to avoid the misleading claim. The revised text reads:

“Extended metrics refer to composite measures derived from space syntax core metrics (integration, connectivity, depth) in combination with other data or analytical tools. Among the 18 included studies, common research methods included combining GPS with Baidu heatmaps. Specifically, combining Baidu heatmaps can be used to reveal the impact of spatial structure on tourist clustering distribution. For example, Huang and Lee explored the combination of Baidu heatmaps and spatial syntax, finding a significant correlation between integration degree and tourist clustering heat.

Zhang et al. combined spatial syntax with GPS trajectory data to generate specific indicators such as visit rate, average dwell time, average walking speed, and revisit rate using GPS data, they then used Spearman correlation analysis to examine the relationship between GPS indicators and spatial syntax indicators. The results showed that walkable accessibility determines the likelihood of a visitor's first visit, while visual features have a greater influence on a visitor's willingness to revisit. By integrating space syntax metrics with behavioral or perceptual data, extended metrics can systematically reveal associations between spatial characteristics and outcomes such as visitor clustering, satisfaction, and revisit intention. They overcome the limitations of single metrics and, through multi-dimensional data integration, enhance the explanatory and predictive power for the relationship between garden spatial structure and tourist behavior. ”

**10.** I noticed in 4.1 that there is a full stop after the names of authors even if they are just one or two (and not "et al."), for example with "In addition, Lee.[34] elucidated the relationship". Please correct throughout.

**Response:** Thank you for pointing this out. The content related to Lee [34] in Section 4.1 has been removed in this round of optimization and revisions. Nevertheless, in response to your comment, we have made corresponding updates throughout the manuscript.

**11.** In section 6 "Different spatial representations" should have a space prior to the previous full stop.

**Response:** Thank you for your careful observation. We have made the corrections, and after a thorough check, we have updated the entire manuscript accordingly

**Reviewer #4:** PLOS ONE, Manuscript Number: PONE-D-25-34363R1 Title: The Influence of Garden Spatial Configuration on Tourist Behavior: A Systematic Review Based on Space Syntax As a reviewer assessing the revised manuscript (Number: PONE-D-25-34363R1), I am satisfied with the revisions made by the authors. They have addressed all of my previous comments and suggestions comprehensively, which has strengthened the manuscript. The manuscript is now in substantially better shape, and I have no further comments at this stage.

**Response:** Thank you very much for your thoughtful review and positive evaluation. We truly appreciate your time and constructive feedback during the previous round, which helped us strengthen the manuscript. We are grateful that the revised version meets your expectations, and we will maintain the same level of rigor in any subsequent steps.
